# VitisNet: “Omics” Integration through Grapevine Molecular Networks

**DOI:** 10.1371/journal.pone.0008365

**Published:** 2009-12-21

**Authors:** Jérôme Grimplet, Grant R. Cramer, Julie A. Dickerson, Kathy Mathiason, John Van Hemert, Anne Y. Fennell

**Affiliations:** 1 Horticulture, Forestry, Landscape, and Parks Department, South Dakota State University, Brookings, South Dakota, United States of America; 2 Department of Biochemistry, University of Nevada Reno, Reno, Nevada, United States of America; 3 Department of Electrical and Computer Engineering and Bioinformatics and Computational Biology Program, Iowa State University, Ames, Iowa, United States of America; University of Toronto, Canada

## Abstract

**Background:**

Genomic data release for the grapevine has increased exponentially in the last five years. The *Vitis vinifera* genome has been sequenced and *Vitis* EST, transcriptomic, proteomic, and metabolomic tools and data sets continue to be developed. The next critical challenge is to provide biological meaning to this tremendous amount of data by annotating genes and integrating them within their biological context. We have developed and validated a system of Grapevine Molecular Networks (VitisNet).

**Methodology/Principal Findings:**

The sequences from the *Vitis vinifera* (cv. Pinot Noir PN40024) genome sequencing project and ESTs from the *Vitis* genus have been paired and the 39,424 resulting unique sequences have been manually annotated. Among these, 13,145 genes have been assigned to 219 networks. The pathway sets include 88 “Metabolic”, 15 “Genetic Information Processing”, 12 “Environmental Information Processing”, 3 “Cellular Processes”, 21 “Transport”, and 80 “Transcription Factors”. The quantitative data is loaded onto molecular networks, allowing the simultaneous visualization of changes in the transcriptome, proteome, and metabolome for a given experiment.

**Conclusions/Significance:**

VitisNet uses manually annotated networks in SBML or XML format, enabling the integration of large datasets, streamlining biological functional processing, and improving the understanding of dynamic processes in systems biology experiments. VitisNet is grounded in the *Vitis vinifera* genome (currently at 8x coverage) and can be readily updated with subsequent updates of the genome or biochemical discoveries. The molecular network files can be dynamically searched by pathway name or individual genes, proteins, or metabolites through the MetNet Pathway database and web-portal at http://metnet3.vrac.iastate.edu/. All VitisNet files including the manual annotation of the grape genome encompassing pathway names, individual genes, their genome identifier, and chromosome location can be accessed and downloaded from the VitisNet tab at http://vitis-dormancy.sdstate.org.

## Introduction

During the pre-genomics era, gene function was established through a reductionist approach [1] where organism physiology was understood by breaking components into pieces, studying them, and then putting them back together to see the larger picture. With the emergence of genome sequencing, organisms are now seen as complex interactive systems. Systems biology, adapted from the general system theory [2] and the living system theory [3], intends to explain biological phenomena utilizing a systemic view of the objects' relationships rather than their simple composition [4]. Integrative functional genomics combines the molecular components (transcripts, proteins, and metabolites) of an organism and incorporates them into functional networks or models designed to describe the dynamic activities of that organism. While many of the functions of individual parts are unknown or not well defined, their biological role can sometimes be inferred through association with other known parts, providing a better understanding of the biological system as a whole. On a system-wide scale the description requires three levels of information [5], [6]: (1) identification of the components (structural annotation) and characterization of their identity (functional annotation); (2) identification of molecules that interact with each component, which leads to the reconstruction of a biochemical reaction network; and (3) characterization of the behaviors of the transcripts, proteins, and metabolites under various conditions. Integration of the three levels of information into a coherent framework (or canvas) provides a powerful approach to tackle the difficult problem of extracting systems-wide behavior from the component interactions.

The most developed examples of application of this approach can be found in prokaryotes, because of their small genomes [7], [8]. For example, in *E. coli*, 92% of the gene product functions have been experimentally verified. Genome-scale models (GEMs) have been used for metabolic engineering to systematically manipulate *E. coli* strains to overproduce lycopene, lactic acid, ethanol, succinate, amino acids, and many other products including hydrogen and vanillin. New biological discoveries of open reading frames (ORF) can be made by focusing on the gaps in the unknown portions of the Omic maps, using the genomic responses of different genotypes under different conditions to determine the probable gene candidates that fill knowledge gaps. GEMs have been widely used to characterize and understand physiological responses to environmental conditions such as abiotic and biotic stresses. This has been particularly useful in the identification of resistance mechanisms that can be established in new strains.

Such global analyses have become possible with the development of high throughput genomics technologies in both the field of nucleic acid sequencing and quantitative data acquisition. Over the last 20 years, expressed tag sequencing (EST) [9] has been widely utilized for gene discovery and genome characterization. EST data are stored in comprehensive databases such as UniGene [10] or the DFCI Gene Indices [11]. Recently, cheaper and faster Next-Gen sequencing technologies have emerged such as 454 [12] or Illumina [13]. Recently, cheaper and faster Next-Gen sequencing technologies have emerged such as 454 [12] or Illumina [13]. In parallel, methods have been developed for quantitative data acquisition: microarrays are used to quantitatively assess the transcriptome [14]. Two dimensional-gels have routinely been used for proteome studies [15]. Recently, however, gel-free technologies have emerged such as ICAT [16] or iTRAQ [17]. Metabolome studies are performed with a variety of tools such as gas chromatography or high performance liquid chromatography for separation and mass spectrometry and nuclear magnetic resonance for the identification and quantification of the metabolites [18].

Genomics resources for *Vitis vinifera* and related species have proliferated rapidly within the last several years, including EST sequencing [19], [20], [21] to whole genome sequencing [22], [23] and integrated genetic maps [24]. These resources have permitted large-scale mRNA expression profiling studies of gene expression profiles during berry development using cDNA or oligonucleotide microarrays [25], [26]. A high-density, Affymetrix GeneChip® *Vitis vinifera* (Grape) Genome Array containing approximately one-third of the expected gene content of the *V. vinifera* genome with some bias towards leaf and berry tissues was developed, leading to numerous publications [27], [28], [29], [30], [31], [32], [33]. Under the encouragement of the international grape community, the microarray data for several of these experiments has been centralized and can be accessed at PLEXdb (http://www.plexdb.org) [34]. Six additional microarray datasets using cDNA, oligo, or Affymetrix arrays are available through Gene Expression Omnibus (http://www.ncbi.nlm.nih.gov/sites/entrez?db=geo) and citations for publications are also linked to these public data sets [33], [35], [36]. Proteomics resources have also emerged recently. Most of these studies use 2-D gel analysis and focus either on berry metabolism [37], [38] or abiotic stress resistance [39], [40], [41] or both [42]. Recently high resolution techniques, such as iTRAQ, have also been applied to grape [43]. Metabolomics studies for grape are still rudimentary; however, several works have presented simultaneous analysis of about 50 to 120 compounds [28], [30], [42], [44]. To date, only two studies present the transcriptomic, proteomic, and metabolomic analyses on the same material, one in berry tissues [29], [42] and the other on abiotic stress in shoots [40], [28].

Information from structural and functional genomics must be combined with detailed biochemical reaction networks to further our understanding of biological function and incorporate the knowledge into cultural practice. While a considerable amount of effort has been put into resolving the structural information (level 1) and “Omics” characterization of individual groups of transcripts, proteins or metabolites (level 3), relatively few biochemical reaction networks (level 2) have been constructed in grapevines or other plant systems. While pathway databases exist at the KEGG (http://www.genome.jp/kegg/pathway.html) or AraCyc [45], they are limited to metabolic pathways. In contrast, MetNet (http://metnet3.vrac.iastate.edu/) stores both metabolic and regulatory interactions for *Arabidopsis* and soybean [46].

In order to contextualize the molecular structure and a metric representing their behavior, we have developed a model of the molecular networks present in grapevines (VitisNet). This resource allows visualization of the dynamic interactions in the transcriptome, proteome, and metabolome within known molecular networks (for example, metabolic or signaling pathways). Integrating transcripts with protein and metabolite profiles in a comprehensive molecular map enables the researcher to elucidate different biochemical responses of grapevines to developmental and environmental cues.

## Results and Discussion

### A Set of 39,424 Unique Sequences Defined

**Figure 1:**
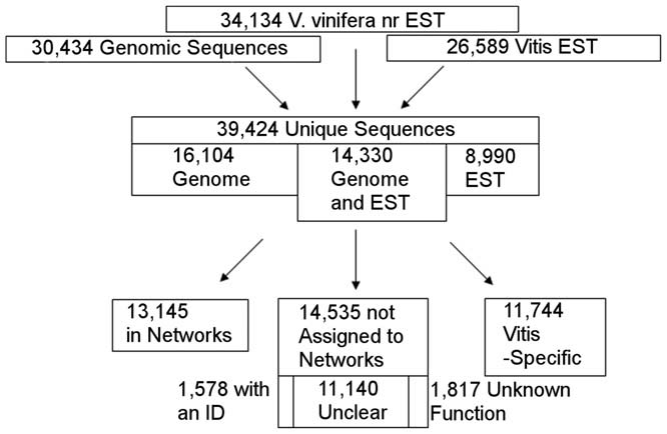
Overview of the unique set assembly and results of the annotation procedure. Box sizes are relative to respective number of genes inside.

The set of unique genes was not restricted to the Pinot Noir genome sequences, as an extensive amount of data have been produced on other *V. vinifera* cultivars and other *Vitis* species. The *V. vinifera* EST database contains only a very small fraction of Pinot Noir sequences (1.8% or 6,385/353,688), whereas Cabernet Sauvignon (half of the EST sequences), Chardonnay, Thompson Seedless, Muscat de Hambourg, and Perlette each have at least two times the number of Pinot Noir sequences. In addition, a significant amount of ESTs have been produced for other *Vitis* species. It is expected that a significant amount of transcript sequences are cultivar and species specific and may not be represented within the Pinot Noir PN40024 genome. A set of 39,424 unique sequences were defined after the matching of the genomic sequences and the transcripts (Figure 1). Only 36.4% of these sequences (14,330) were found in both the genomic sequences and the transcripts. In the set of unique sequences, the genomic sequences were conserved over transcript sequences because they should be the full length gene, whereas there is less certainty for the transcript. In some cases, several supposedly unique transcript sequences matched a single gene, mainly because they matched different regions of the gene. A total of 652 unique sequences corresponded to previously published grapevine sequences (Table S1).

The set that was found only in the genomic sequences included 40.8% (16,104) of the unique sequences. This means that so far there is no proof that these sequences are actually transcribed. Finally 22.8% (8990) of the unique sequences were found only as transcripts. This set could include cultivar or species specific genes absent in the Pinot Noir genome or genes not yet extracted from the genome. However as 73% (6553) of these unique sequences were not homologous to sequences from other organisms, it is likely that most of them corresponded to short sequences or contained mostly UTR regions so that a BLAST analysis could not be conducted against the genome sequences encoding for their putative proteins. These sequences were of interest because many of them were placed on the highly popular Affymetrix GeneChip® *Vitis vinifera* (Grape) Genome Array. There were 3208 sequences amongst the 11,734 non-redundant sequences in the Affymetrix chip that did not present a match in the genome.

### Half of the Matched Sequences Were Assigned to Molecular Networks

Seventy percent (27,680) of the unique genes matched a previously described *Vitis* cDNA or protein or a sequence from another organism. The remaining 11,744 sequences were *Vitis*-specific and a function could not be assigned. This number rose to 83% when only genes from the genome sequences were used. This gene set was divided into two groups, a group that could not be assigned to molecular networks and a group that could be assigned. The group that was not assigned to molecular networks consisted of 14,535 genes (52.5%) that covered a wide range of functional descriptions. At one extreme, the sequences (1,817) presented a completely unknown function. At the other extreme, an identifier was attributed to unmapped sequences (1,578). An identifier was assigned because an EC or KO number could be attributed to these sequences or an *Arabidopsis* homolog had an identifier; however, they couldn't be placed on the networks. In between the unknown and EC/KO identity, the description of the function ranged from sequences containing a poorly described domain, a general enzymatic activity, or to a well-documented gene.

The second subset of the matched genes (13,145 sequences, 47.5%), which were homologous to proteins with a known function, was assigned to the molecular networks. The 13,145 genes present in the networks were classified into 6 main overlapping categories (Table 1-6): Metabolism (5442 sequences), Genetic Information Processing (1249 sequences), Environmental Information Processing (1305 sequences), Cellular Processes (1121 sequences), Transport (3523 sequences), and Transcription Factors (2423 sequences). The complete annotation of the genes and relevant information for each is presented in Table S1. The references used for annotating genes and for developing pathways not found in KEGG are presented in Text S1.

**Table 1:** List of Metabolic Pathways.

VVID	Network Name	#gen	#pro	#met	VVID	Network Name	#gen	#pro	#met	
**1.1**	**Carbohydrate Metabolism**									
10010	Glycolysis / Gluconeogenesis	192	28	28	10530	Aminosugars metabolism	90	9	11	
10020	Citrate cycle (TCA cycle)	74	17	21	10520	Nucleotide sugars met.	60	16	18	
10030	Pentose phosphate pathway	83	17	21	10620	Pyruvate metabolism	197	27	19	
10040	Pentose/glucuron. interconv.	57	11	14	10630	Glyoxyl., dicarboxyl. met.	90	18	19	
10051	Fructose and mannose met.	108	21	21	10640	Propanoate metabolism	73	9	12	
10052	Galactose metabolism	155	17	27	10650	Butanoate metabolism	85	18	22	
10053	Ascorbate and aldarate met.	40	9	9	10562	Inositol phosphate met.	131	18	20	
10500	Starch and sucrose met.	337	43	34						
**1.2**	**Energy Metabolism**									
10190	Oxidative phosphorylation	343	101	7	10720	Red. Carb. cyc. (CO2 fix.)	41	10	14	
10195	Photosynthesis	173	52		10680	Methane metabolism	130	9	11	
10196	Photosynthesis - antenna prot.	27	11		10910	Nitrogen metabolism	112	22	19	
10710	Carbon fixation	140	21	20	10920	Sulfur metabolism	46	12	12	
**1.3**	**Lipid Metabolism**									
10061	Fatty acid biosynthesis	76	13	36	10561	Glycerolipid met.	146	19	18	
10062	Fatty acid elongation in mitoc.	25	7	29	10564	Glycerophospholipid met.	140	29	32	
10071	Fatty acid metabolism	94	17	40	10565	Ether lipid metabolism	57	8	9	
10072	Synth. / degr. of ketone bodies	18	3	4	10600	Sphingolipid metabolism	67	13	15	
10100	Biosynthesis of steroids	142	47	74	10592	alpha-Linolenic acid met.	104	14	29	
10140	C21-Steroid hormone met.	20	6	14	11040	Biosynth. unsat. fatty ac.	42	14	27	
**1.4**	**Nucleotide Metabolism**									
10230	Purine metabolism	151	48	62	10240	Pyrimidine metabolism	109	35	46	
**1.5**	**Amino Acid Metabolism**									
10251	Glutamate metabolism	93	28	25	10330	Arginine and proline met.	54	17	23	
10252	Alanine and aspartate met.	109	23	24	10340	Histidine metabolism	70	16	19	
10260	Gly, ser and thr met.	110	30	38	10350	Tyrosine metabolism	149	25	39	
10271	Methionine metabolism	124	33	48	10360	Phenylalanine metabolism	212	15	14	
10272	Cysteine metabolism	78	17	25	10380	Tryptophan metabolism	20	6	7	
10280	Val, leu and Ile degr.	85	18	34	10400	Phe, tyr and try biosynth.	144	30	35	
10290	Val, leu and Ile biosynth.	60	13	26	10220	Urea cyc., met. amino grp	120	31	41	
10300	Lysine biosynthesis	82	17	22						
**1.6**	**Met. of Other Amino Acids**									
10410	beta-Alanine met.	60	12	13	10460	Cyanoamino acid met.	35	8	16	
10450	Selenoamino acid met.	69	15	17	10480	Glutathione met.	127	35	16	
**1.7**	**Glycan Biosynth. And Met.**									
10510	N-Glycan biosynthesis	50	19	21	10563	GPI-anchor biosynthesis	21	12	14	
10511	N-Glycan degradation	67	8		10602	Glycosphingolip. biosynth.	15	7	16	
10540	Lipopolysac. Biosynth.	12	10	13	11030	Glycan struct. biosynth. 1	88	26	49	
10550	Peptidoglycan biosynth.	18	3	15						
**1.8**	**Met. of Cofactors and Vit.**									
10730	Thiamine metabolism	21	12	20	10780	Biotin metabolism	12	6	8	
10740	Riboflavin metabolism	63	12	15	10790	Folate biosynthesis	39	18	24	
10750	Vitamin B6 metabolism	23	7	13	10670	One carbon pool by folate	42	15	9	
10760	Nicotinate, nicotinamide met	30	12	13	10860	Porph. and chloroph. met.	67	31	39	
10770	Pantothenate, CoA biosynth.	44	15	19	10130	Ubiquinone biosynthesis	31	17	25	
**1.9**	**Biosynth. of Secondary Met.**									
10900	Terpenoid biosynthesis	182	18	24	10941	Flavonoid biosynthesis	183	25	52	
10904	Diterpenoid biosynthesis	72	18	37	10942	Anthocyanin biosynthesis	59	8	18	
10902	Monoterpenoid biosynth.	192	24	37	10943	Isoflavonoid biosynthesis	63	7	17	
10908	Zeatin biosynthesis	52	10	20	10950	Alkaloid biosynthesis I	65	17	23	
10906	Carotenoid biosynth.	40	19	33	10311	Penicillin/cephalosp. bioS.	14	4	5	
10905	Brassinosteroid biosynth.	19	7	24	11002	Auxin biosynthesis	98	18	12	
10940	Phenylpropanoid biosynth.	220	21	44	11012	IBA metabolism	14	11	5	
**1.10**	**Other**									
11000	Single reactions	162	15	38						

VVID: VitisNet identification number; #gen: number of genes in network; #pro: number of proteins in network; #met: number of metabolites in network.

**Table 2 pone-0008365-t002:** List of Genetic Information Processing Networks.

VVID	Network Name	#gen	#pro	VVID	Network Name	#gen	#pro	#met
**2.1**	**Transcription**							
23020	RNA polymerase	85	32	23022	Basal transcription factors	55	20	
**2.2**	**Translation**							
23010	Ribosome	473	147	20970	Aminoacyl-tRNA biosynthesis	128	22	64
**2.3**	**Folding, Sorting Degr.**							
23060	Protein export	36	16	24120	Ubiquitin mediated proteolysis	158	65	4
24130	SNARE int. in ves. transport	63	22	24140	Regulation of autophagy	48	15	5
23050	Proteasome	58	48					
**2.4**	**Replication and Repair**							
23030	DNA replication	60	38	23430	Mismatch repair	37	19	
23410	Base excision repair	31	21	23440	Homologous recombination	39	19	
23420	Nucleotide excision repair	53	36	23450	Non-homologous end-joining	14	8	

VVID: VitisNet identification number; #gen: number of genes in network; #pro: number of proteins in network; #met: number of metabolites in network.

**Table 3 pone-0008365-t003:** List of Environmental Information Processing Networks.

VVID	Network Name	#gen	#pro	#met	VVID	Network Name	#gen	#pro	#met
**3.1**	**Signal Transduction**								
34020	Calcium signaling	142	27	22	34150	mTOR signaling	28	17	
34070	Phosphatidylinositol sign. syst.	98	13	17					
**3.2**	**Hormone Signaling**								
30001	ABA signaling	102	56	11	30008	Ethylene signaling	248	101	3
30003	Auxin signaling	262	103	2	30010	Gibberellin signaling	31	14	1
30005	Brassinosteroids signaling	30	13	2	30011	Jasmonate signaling	86	36	4
30007	Cytokinin signaling	70	42	2					
**3.3**	**Plant-Specific Signaling**								
34710	Circadian rhythm	94	48		30009	Flower development	185		

VVID: VitisNet identification number; #gen: number of genes in network; #pro: number of proteins in network; #met: number of metabolites in network.

**Table 4 pone-0008365-t004:** List of Cellular Processes Networks.

VVID	Network Name	#gen	#pro	#met
**4.1**	**Cell Motility**			
44810	Regulation of actin cytoskeleton	360	114	1
**4.2**	**Cell Growth and Death**			
44110	Cell cycle	315	192	
**4.3**	**Cell Wall**			
40006	Cell wall	448	53	11

VVID: VitisNet identification number; #gen: number of genes in network; #pro: number of proteins in network; #met: number of metabolites in network.

**Table 5 pone-0008365-t005:** List of Transport Networks.

VVID	Network Name	#gen	#pro	#met.	VVID	Network Name	#gen	#pro
**5.1**	**Membrane Transport**							
52010	ABC transporters	283	87					
**5.2**	**Hormone Transport**							
50004	Auxin transport	57	23	2				
**5.3**	**Transport System**							
50110	Protein coat	157	83		50112	Nuclear pore complex	72	26
50111	tethering factors	100	65		50113	Thylakoid targeting pathway	62	15
**5.4**	**Transporter Catalog**							
50101	Channels and pores	391	131		50124	Porters categories 30 to 64	155	69
50104	Group translocators	39	4		50125	Porters categories 66 to 94	215	50
50105	Transport electron carriers	89	38		50131	Prim. active transp. cat. A2-A4	200	44
50108	Accessory fact. Inv. in transp.	173	11		50132	Prim. active transp. cat. A5-A8	184	69
50109	Incomp. charact. transp. syst.	332	101		50133	Prim. act. transp. cat. A9-A18	191	71
50121	Porters categories 1 to 6	187	85		50134	Primary. active transp. Cat. D1	164	39
50122	Porters categories 7 to 17	242	49		50135	Prim. active transp. Cat. D3-E2	125	43
50123	Porters categories 18 to 29	204	46					

VVID: VitisNet identification number; #gen: number of genes in network; #pro: number of proteins in network; #met: number of metabolites in network.

**Table 6:** List of Transcription Factors Networks.

VVID	Network Name	#gen	#pro	VVID	Network Name	#gen	#pro	VVID	Network Name	#gen	#pro
60001	ABI3VP1	26	26	60028	FHA	19	19	60055	SBP	22	22
60002	Alfin	8	8	60029	G2-like	39	39	60056	SET PCG	52	52
60003	AP2 EREBP	139	139	60030	GeBP	7	7	60057	Sigma70-like	8	8
60004	ARF	27	27	60031	GIF	4	4	60058	SNF2	44	44
60005	ARID	11	11	60032	GRAS	53	53	60059	SRS	5	5
60006	ARR-B	15	15	60033	GRF	14	14	60060	TAZ	7	7
60007	AS2	42	42	60034	HB	93	93	60061	TCP	20	20
60008	AUXIAA	28	28	60035	HMG	16	16	60062	Trihelix	36	36
60009	BBR	5	5	60036	HRT	1	1	60063	TUB	17	17
60010	BES1	7	7	60037	HSF	23	23	60064	ULT	1	1
60011	BHLH	146	146	60038	Jumonji	27	27	60065	VOZ	2	2
60012	BZIP	66	66	60039	LFY	1	1	60066	WRKY	69	69
60013	BHSH	1	1	60040	LIM	15	15	60067	zf-MYND	4	4
60014	C2C2-CO	15	15	60041	LUG	7	7	60068	zf-HD	15	15
60015	C2C2-DOF	26	26	60042	MADS	71	71	60069	ZIM	14	14
60016	C2C2-GATA	20	20	60043	MBF1	5	5	60070	Orph_CCT	9	9
60017	C2H2	117	116	60044	MYB	176	176	60071	Orph_FAR-RED	53	53
60018	C3H	79	79	60045	MYB rel.	59	59	60072	Orph_Resp_reg	14	14
60019	C2C2-YABBY	7	7	60046	NAC	86	86	60073	Orph_zf-b_box	14	14
60020	CAMTA	6	6	60047	PBF-2like	2	2	60074	Orph_zf-SWIM	9	9
60021	CCAAT	30	30	60048	PHD	71	71	60075	Other BSD	8	8
60022	CPP	7	7	60049	PLATZ	11	11	60076	Other GTF	7	7
60023	CSD	3	3	60050	PsARR-B	8	8	60077	Other zf-AN1	14	14
60024	DBP	3	3	60051	RB	2	2	60078	Other zf-C3HC4	244	244
60025	DDT	8	8	60052	RWP-RK	10	10	60079	Other zf-DHHC	24	24
60026	E2F-DP	9	9	60053	S1Fa-like	3	3	60080	Other zf	32	32
60027	EIL	4	4	60054	SAP	1	1				

VVID: VitisNet identification number; #gen: number of genes in network; #pro: number of proteins in network.

### Construction of 219 Networks

**Figure 2:**
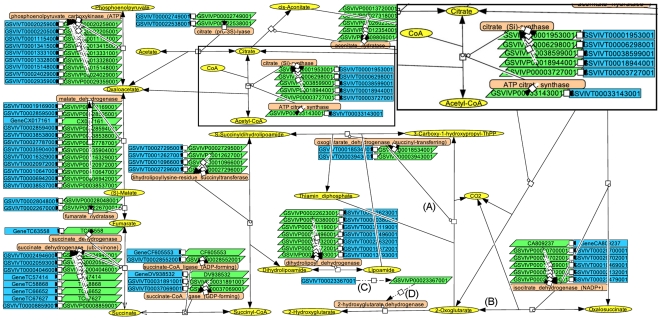
Citrate cycle pathway visualized using CellDesigner. Symbols represent different molecules or reactions, i.e. blue rectangle: gene; green parallelogram: transcript; orange round rectangle: protein; and yellow ellipse: metabolite. Edges with a circle at the tip: catalysis (A). Edges with Delta at the tip: metabolic reaction (B). Edges with dash-dot-dot-dash: transcription (C). Edges with dash-dot-dash: translation (D). Insert box at the upper right represents a zoom-in of an area of the network showing the different molecule types.

**Figure 3:**
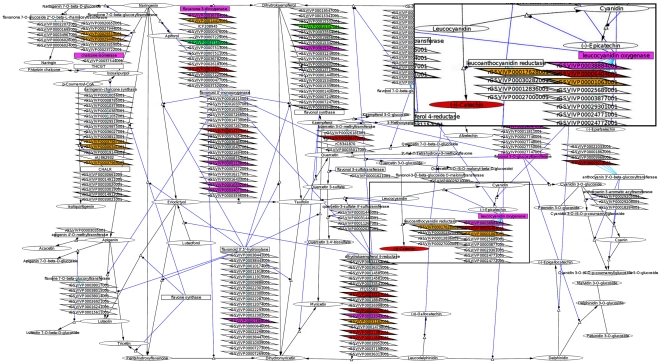
Flavonoid biosynthesis pathway and tissue-specific molecule abundance visualized using Cytoscape. Parallelogram: transcript; rectangle: protein; ellipse: metabolite; triangle: reaction node. Blue edges with circle at the tip: catalysis. Black edges with Delta at the tip: metabolic reaction. Turquoise edges: translation. Transcript node in bold: existence of an Affymetrix (*Vitis Vinifera* (Grape) Genome Array) probeset. Red: over abundant in seed; magenta: over abundant in skin; green: over abundant in pulp; orange: over abundant in seed and skin. Insert box at the upper right represents a zoom-in of an area of the network showing the different molecule types.

The networks were constructed with the CellDesigner software. This software has the benefit of being able to save the networks in the SBML (System Biology Markup Language) format. This format is highly portable into a variety of software packages, including Cytoscape, which was used here for data visualization of molecular expression. The networks were constructed with four main families of nodes (gene, transcripts, proteins, and metabolites) represented by specific shapes and colors in CellDesigner (Figure 2) and by shape only in Cytoscape (Figure 3; color was used to visualize abundance). In VitisNet, some extra node styles can be used in the networks for additional categories (phenotypes, phylogenic tree node, etc.). Edge styles represented different types of reactions, and they were specified by shape in CellDesigner and color in Cytoscape; Text S2 has a legend that summarizes the node and edge styles used in VitisNet in Cytoscape. Five digit IDs were assigned to the networks (Table S2). The first digit refers to the network category (metabolic pathway etc.), and the last four digits refer to the KEGG pathway number (if it existed in KEGG).

#### Metabolic pathways (1)

Metabolic pathways are the most common type of pathway that can be found for plants in several online databases such as KEGG or PlantCyc (http://www.plantcyc.org/). These networks (Table 1) represented metabolic reactions known to occur in grapevines. With the software package KEGG2SBML, it was easy to import the metabolic pathways from KEGG. The KEGG pathways were limited when they were used; they only showed metabolites and proteins involved in reactions and included reactions that may not occur in plants. Therefore, additional information and symbols representing the missing grape genes and transcripts were added to the networks in VitisNet described in this paper. Reactions in KEGG without a putative grape protein identified and for which no evidence for their presence in plants could be found in the literature were removed. Finally, reactions in grapevines that were absent in KEGG were manually added to the networks. The total number of items in the 88 grape metabolic pathways constructed included: 7,854 genes and transcripts, 1,631 proteins, and 1,998 metabolites. Some of these items were present in more than one network.

#### Genetic information processing (2)

The category “Genetic Information Processing” (Table 2) corresponds to housekeeping mechanisms that are present and highly conserved in all eukaryotes. These networks were present on the KEGG website but in a different format than the metabolic networks; therefore exportation with KEGG2SBML was not possible. These networks were represented by a picture of a specific *modus operandi*, with every involved protein listed at the side rather than in a diagram of the enzymatic reactions. In VitisNet, we have tried to represent these pictures interactively. Where this was not possible, the networks were presented as lists of genes, transcripts, and proteins. The total number of items in the 15 “Genetic Information Processing” networks included 1,338 genes and transcripts, 527 proteins, and 71 metabolites.

#### Environmental information processing (3)

The category “Environmental Information Processing” (Table 3) represents signal processes that occur in the grapevine. The networks belonging to “Signal Transduction” are highly variable amongst species but they are well documented for *Arabidopsis* in KEGG and were constructed using the *Arabidopsis* data. The networks for hormone signaling and plant-specific signaling were reconstructed from the literature. To the best of our knowledge, these networks could not be found in any other pathway databases. These networks are particularly valuable for the plant community since hormonal signaling is an important subject in many plant physiology studies. The total number of items in the 12 “Environmental Information Processing” networks included 1,373 genes and transcripts, 563 proteins, and 63 metabolites.

#### Cellular processes (4)

These networks for the “Cellular Processes” category (Table 4) were named from the KEGG pathways; however the KEGG pathways were not related to the molecular events occurring in plants. Although a small portion of the pathways were derived from KEGG, most components of the networks were constructed from information collected from the literature. The total number of items in the 3 “Cellular process” networks included 1,123 genes and transcripts, 359 proteins, and 12 metabolites.

#### Transport (5)

The networks for Hormone Transport (5.2) and Transport Systems were constructed from the literature (Table 5). The networks in “Transporters Catalog” present the classification of the putative grape transporters according to the transporter classification (TC) system. This classification was formally adopted by the International Union of Biochemistry and Molecular Biology (IUBMB) in June 2001 and is the international standard for the classification of transporters. In VitisNet, molecules designating a transporter were linked to their corresponding category. The total number of items in the 21 “Transport” networks included 3,622 genes and transcripts, 1,149 proteins, and 1 metabolite.

#### Transcription factors (6)

These networks presented the classification of the grape putative transcription factors (Table 6). The classification used here was a customized version of two plant transcription factor databases that contained a total of 80 families. The PlantTFDB [47] contained 64 families and the PlnTFDB [48] contained 68 families. Most of the families (58) were present in the two databases, although two families were exclusive to PlantTFDB and eight were exclusive to PlnTFDB. In addition, 12 families were exclusive to the grapevine transcription factors. Representatives of five of these families were present in the plntfdb under the family named “orphans” and we chose to break this group into distinct families. The seven other families identified were proteins that contain a domain found in BTF2-like transcription factors, Synapse associated proteins and DOS2-like proteins (BSD, [49]), the Global Transcription Factor group (GTF), and subfamilies of zinc finger proteins. The transcription factor families were presented as a phylogenetic tree, which allowed subfamilies to be grouped together. The total number of items in the 80 “Transcription factors” networks included 2,423 genes, transcripts, and proteins.

### Omics Data Can Be Visualized on the Networks

Annotation of the genes and construction of VitisNet has filled a major gap in precise descriptive and quantitative tools for grapevine systems biology. The next challenge is the integration of the data. The molecular networks were built to allow simultaneous visualization of transcripts, proteins, and metabolites. Their respective abundance under various conditions can be visualized through the Cytoscape software.

Several methods exist to correlate and integrate transcript, protein, and metabolite profiles. For example molecular abundance profiles were linked with Pearson [50], [51] and Spearman [52] correlation coefficients, the BL-SOM method [53], [54] and the O2PLS method [55]. The O2PLS method enables the determination of the effect of each variable, in a multivariable experiment, on the co-expression of molecules. More recently the O2PLS method has been developed further to integrate all three molecular profiles (transcripts, proteins, and metabolites) [56].

In most of these statistical studies, data were visualized by representing molecules by nodes and the correlation by edges. Subsequently, selected pathways were drawn manually for biological phenomenon highlighted by the correlations of molecular abundance. In the visualization of “omics” data in VitisNet, edges represented biological processes and nodes represented molecules, as in classical presentations of pathways. Molecular abundance was represented by color changes of the nodes and biological phenomenon could be visualized automatically. As an illustration of the methodology used in VitisNet to provide visualization of “omics” data, datasets from a study of the differential transcript, protein, and metabolite abundance measured in three berry tissues [29], [42] was uploaded into the molecular maps. For consistency, proteins and metabolites [42] were clustered with the same methods used for clustering the transcripts [29] and the same color scheme was used, (green = molecules over-abundant in pulp, purple = molecules over-abundant in the skin, and orange = molecules over-abundant in seed [29]). The flavonoid biosynthesis pathway (Figure 3) presented here was more complex than previous representations of the pathway in [29] and [42]. Here it was further customized from the total flavonoid biosynthesis pathway in VitisNet by removing the gene nodes for easier visualization. As these studies have illustrated, molecules involved in the flavonoid biosynthesis pathway are slightly more abundant in skin than seed and clearly more abundant in both skin and seed than in the pulp. Transcriptomic results from Affymetrix GeneChip® *Vitis vinifera* (Grape) Genome Array were used here, but data from any microarray platform can be uploaded onto the networks. For example, Table S1 contains data for mapping the cDNA array used in a grape bud chilling requirement fulfillment study [35]. The integration of the berry tissues “omic” data on all the pathways was divided into higher level pathway categories; the Cytoscape session files, molecular networks and a tutorial (Text S2) can be accessed and downloaded at the VitisNet tab at vitis-dormancy.sdstate.org. All molecular network files are also available for browsing or downloading at MetNet (http://metnet3.vrac.iastate.edu/)

### Conclusion

An exhaustive coverage of the network of grapevine molecules has been developed. It presents an easy, fast, and comprehensive method for simultaneous integration and visualization of “omics” data. These molecular networks provide biological value for both grapevine researchers and the rest of the plant scientist community. The following attributes are provided: (i) original plant-specific pathways within VitisNet, (ii) the possibility to create a mapping file of genes from other plants, and (iii) the ability to customize the schematics for new or species-specific reactions. In the future, in cooperation with the scientific community's curation of gene annotations, we are planning to release new networks and update existing networks with emerging data (ie. miRNA) at MetNet (http://metnet3.vrac.iastate.edu/) and VitisNet (http://vitis-dormancy.sdstate.org/pathways.cfm).

## Materials and Methods

### Definition of a Unique Set of Genes

The 30,434 DNA sequences encoding for putative proteins from the *Vitis vinifera* (c.v Pinot noir PN40024) genome [23] were matched to EST sequences from *Vitis vinifera* and other *Vitis* species. The *V. vinifera* sequences originated from the 5.0 release of the DFCI grape index (http://compbio.dfci.harvard.edu/tgi/cgi-bin/tgi/gimain.pl?gudb=grape) which contained 34,134 unique sequences. The set of non-vinifera sequences contained a total of 26,589 redundant ESTs obtained from the NCBI website. This set included sequences from the following species: *V. shuttleworthii* (10,704 sequences), hybrid cultivars (6,542 sequences), *V. arizonica x rupestris (*5,421 sequences), *V. aestivalis* (2,101 sequences), and *V. riparia (*1,821 sequences). A BLAST analysis of the sequences from the *V. vinifera* EST set and the non-vinifera EST set (Megablast, p > 95, e-value<1e-15) was conducted against the genomic sequences. Sequences not identified in the genome were added to the genomic sequences to constitute the unique sequences set. The 1395 mRNAs corresponding to grapevine protein sequences registered in UniProt and not belonging to one of the two genome sequencing projects were manually retrieved and BLAST analyzed (blastn e-value <1 e-15) against the unique sequences set.

### Gene Annotation

During the first steps of annotation, a batch BLAST analysis (blastx, e-value<1e-10) of unique sequences was conducted against several relevant databases, including the *Arabidopsis* and rice genomes and the *Viridiplantae* protein sequences in NCBI. For each gene, the ten best significant matches in each database were conserved and reviewed for defining the most likely annotation. Particular attention was paid to using identical nomenclature for genes with the same function. A BLAST analysis of the genes that had at least one significant match containing a putative function was conducted against the KEGG database (http://www.genome.jp/kegg/) for defining an enzyme commission (EC) number or a KEGG Orthology (KO) number. For genes not identified in this screen, the EC number of genes suspected to encode for a protein with enzymatic function was identified by browsing enzyme nomenclature databases (such as Expasy (http://www.expasy.org/enzyme/) or BRENDA (http://www.brenda-enzymes.org/)). A BLAST analysis (blastx, e-value<1e-10) of the unique set was conducted against the Transport Classification Database (TCDB) (http://www.tcdb.org/) and the genes matching sequences from that database were again manually reviewed and assigned to a category from the Transport Classification System [57].

BLAST analysis (blastx, e-value<1e-10) of the unique set was conducted against two plant transcription factor databases, PlantTFDB, (http://planttfdb.cbi.pku.edu.cn/) [47] and PlnTFDB (http://plntfdb.bio.uni-potsdam.de/v2.0/) [48]. InterPro domains obtained for the grape sequences from the UniProt website were also used for the classification of transcription factors. The transcription factors were then grouped into families.

Where molecular interactions were identified in the literature, the gene function was browsed to identify the *Vitis* gene potentially involved. The genes described in the literature were validated by BLAST against the unique set of *Vitis* sequences to correctly identify any potential homolog that was previously mislabeled.

A short identifier was defined for genes that were present on the networks but did not have a previously defined EC number or a KO. For most of these, that identifier corresponded to the one commonly used for their *Arabidopsis* homolog in their Entrez webpage (http://www.ncbi.nlm.nih.gov/sites/entrez?db=gene). For genes without an *Arabidopsis* homolog with a clear identifier, a unique identifier was created that was consistent with the gene function.

### Network Construction

#### Metabolic pathways (1)

KEGG metabolic pathways were downloaded from the KEGG website and converted into SBML files with the KEGG2SBML software package [58]. Grape genes and transcripts were manually added to the networks and linked to their corresponding proteins with the CellDesigner software package [59]. Plant- or grape-specific reactions that were not present in KEGG but were described in the literature were added manually.

#### Genetic information processing (2), signal transduction (3.1), and ABC transporters (5.2)

KEGG pathways were manually reconstructed with CellDesigner using the SBML format, and then grape genes and transcripts were manually added to the networks and linked to their corresponding proteins. Plant- or grape-specific processes that were not present in KEGG but were described in the literature were manually added.

#### Hormones signaling (3.2), plant-specific signaling (3.3), cellular processes (4), hormone transport (5.2), and transport system (5.3)

Networks were manually constructed from the literature with CellDesigner using the SBML format, and then grape genes and transcripts were manually added to the networks and linked to their corresponding proteins.

#### Transport catalog (5.4)

Networks were manually constructed with CellDesigner using the SBML format. Grape genes and transcripts matching transporter proteins from any other organisms were manually added to the networks and linked to their corresponding proteins. Proteins were linked to an object class representing a transporter subcategory from the TCdb.

#### Transcription (6)

Networks were manually constructed with CellDesigner using the SBML format. Grape genes and transcripts matching transcription factors from other species were manually added to the networks and linked to their corresponding proteins. For each transcription factor family, a phylogenetic tree was constructed based on protein alignment generated with the neighbor-joining method using ClustalW. The transcription factors were then grouped according to the phylogenetic tree. Distances are not related to respective phylogenic distances. All the relevant bibliography for the construction of literature-based pathways is included in Table S2 and Text S1.

### Expression Profiling

Affymetrix probesets were matched to the genome using the same process as that used between the genome sequences and EST sequences. The tentative contigs from the DFCI Grape Gene Index (http://compbio.dfci.harvard.edu/tgi/cgi-bin/tgi/gimain.pl?gudb=grape), that contain the ESTs that were used as templates for the Affymetrix probesets, were BLAST analyzed against the genome sequences (Megablast, p>95, e-value<1e-15).

Transcriptomic data were retrieved from Grimplet et al. [29]. Proteomics and metabolomics data were retrieved from Grimplet et al. [42]. All molecules with differential abundance were grouped into 12 clusters presented by Grimplet et al. [29] according to their abundance in the three berry tissues. Data were visualized using VitisNet with the Cytoscape software [60] (see Text S2 for a tutorial on the complete procedure).

## Supporting Information

Table S1 The complete grape gene annotation based on the 8X assembly (Jaillon et al., 2007) of transcript sequences. Unique Gene: Genoscope ID (Jaillon et al., 2007) is used if a genome sequence has been identified, otherwise VVGI 5 TC (Tentative Consensus sequences) number or EST GenBank ID is used. Unique transcript: VVGI 5 TC number or EST GenBank ID is used if a transcript has been identified, otherwise the Genoscope ID is used. Function: tentative functional annotation. Network ID: the identifier that is used in the networks. Network or simplified category: list of the networks where the genes appear, otherwise a short description of the biological role. In Network: the gene is present in at least one network. Probeset: probeset ID for the Affymetrix GeneChip® Vitis vinifera (Grape) Genome Array. Best Arabidopsis match: best matched hit in Arabidopsis putative proteins. InterPro domain ID: list of the domains detected from InterPro (Hunter et al., 2009). Gene Ontology ID: list of the identified GO terms. Gene Ontology description: description of the GO term (The Gene Ontology Consortium, 2009). Accession UniProt: UniProt ID for the genome sequences (Apweiler et al., 2004). Accession UniProt for published grapevine protein: UniProt ID for grapevine proteins individually published apart of the genome sequencing. EST probeset: EST from which the probeset was designed. IASMA gene: ID from the heterozygote Vitis genome (Velasco et al., 2007). Chromosome position: position of the gene on chromosome retrieved from Gramene.org. Other Vitis: presence in non-vinifera Vitis species. cDNA array: ID used in the cDNA array from Mathiason et al., (2009). Other TC from VVGI5: list of other TC from the DFCI matching the gene. Other probesets: other Affymetrix probesets matching the gene.
(10.28 MB XLS)

Table S2 List of pathways constructed from bibliographic data and the corresponding journal articles used.
(0.03 MB DOC)

Text S1 References for supporting material.
(0.06 MB DOC)

Text S2 Tutorial for Using VitisNet, a database for the grapevine molecular networks.
(3.51 MB DOC)
