# Supplementary material for: VitisNet: “Omics” Integration through Grapevine Molecular Networks
Source: PLoS One. 2009 Dec 21;4(12):e8365. doi: 10.1371/journal.pone.0008365 (PMC2791446; doi:10.1371/journal.pone.0008365)
Supplement: Table S2 — List of pathways constructed from bibliographic data and the corresponding journal articles used. (0.03 MB DOC) [file pone.0008365.s002.doc]

**Supporting Table 2**: List of pathways constructed from bibliographic data and the corresponding journal articles used.

| **VVID** | | **Pathways** | **Journal Articles** | | |
| --- | --- | --- | --- | --- | --- |
| vv30001 | ABA signaling | | Brocard-Gifford et al., 2004  Chinnusamy et al., 2004  Choi et al., 2005  He and Gan, 2004  Himmelbach et al., 2002 | Hugouvieux et al., 2002  Kariola et al., 2006  Nishimura et al., 2005  Reyes et al., 2007  Riera et al., 2006 | Saez et al., 2006  Stone et al., 2006  Xiong et al., 2002  Yoshida et al., 2006  Zhang et al., 2005 |
| vv30008 | Ethylene signaling | | Adams-Phillips et al., 2004 | Aeschbacher et al., 1995  Guo et al., 2008 | Guo and Ecker, 2004 |
| vv30003 | Auxin signaling | | Cosgrove et al., 2002  Laskowski et al., 2002  Paciorek and Friml, 2006 | Quint and Gray, 2006  Sieberer et al., 2003  Stafstrom et al., 1998 | Woodward et al., 2005  Woodward and Bartel, 2005 |
| vv30010 | Gibberellin signaling | | Aubert et al., 1998  Day et al., 2004 | Dill et al., 2004  Segura et al., 1999 | Silverstone et al., 2007  Tanaka et al., 2007 |
| vv30005 | Brassino-steroid signaling | | Wang et al., 2006 |  |  |
| vv30011 | Jasmonate signaling | | Brodersen et al., 2006  Chung and Howe, 2009  Coego et al., 2005  Dreher et al., 2007  Durrant et al., 2007 | Ellis et al., 2002  Guranowski et al., 2007  Huang et al., 2005  Kang et al., 2003 | Katsir et al., 2008  Nishimura et al., 2005  Schenk et al., 2005  Spoel et al., 2003 |
| vv30007 | Cytokinin signaling | | Kakimoto, 2003  Kimura et al., 2001 | Naito et al., 2007  Sieberer et al., 2003 | Teramoto et al., 1994 |
| vv30009 | Flower development | | Baumann et al., 2007  Ben-Naim et al., 2006  Bezerra et al., 2004  Bowman et al., 1992  Cheng et al., 2003  Cockram et al., 2007  Costa et al., 2005  Ge et al., 2000  He et al., 2004  Hileman et al., 2003  Hord et al., 2006  Jack, 2002  Jaeger et al., 2006 | Jin et al., 2008  Jung et al., 2007  Kania et al., 1997  Kim et al., 2006  Kim and Michaels, 2006  Lee et al., 2006  Lee et al., 2007  Lim et al., 2004  Liu and Meyerowitz, 1995  March-Díaz et al., 2007  Mayama et al., 2003  Michaels et al., 2003  Murtas et al., 2003 | Noh et al., 2004  Parcy et al., 2002  Peng et al., 2006  Robles and Pelaz, 2005  Schmitz et al., 2005  Sridhar et al., 2006  Telfer and Poethig, 1998  Wagner and Meyerowitz, 2002  Wang and Chen, 2004  Yang et al., 1995  Yoshida et al., 2001  Zhao et al., 1999 |
| vv44810 | Reg. of actin cytoskeleton | | Hussey et al., 2006  Kandasamy et al., 2004 | Pilpel and Segal, 2005  Schenck et al., 2004 | Wasteneys and Yang, 2004 |
| vv44110 | Cell cycle | | Azumi et al., 2002  Cui et al., 2007  Francis and Dennis, 2007 | Haga et al., 2007  Ishida et al., 2007  Jiang et al., 2007 | Li et al., 2006  Miyagishima et al., 2006  Rossi and Varotto, 2002 |
| vv50110 | Protein coat | | Hughes and Stephens, 2008 | Nickel et al., 2002 | Swennen and Beckerich, 2007 |
| vv50112 | Nuclear pore complex | | Cole, 1998 |  |  |
| vv50111 | Tethering factors | | Gruenberg and Stenmark, 2004 | Hála et al., 2008  Hurley and Emr, 2006 | Sato, 2000  Swennen and Beckerich, 2007 |
| vv50113 | Thylakoid targeting | | Di Cola et al., 2005 |  |  |
