# Supplementary material for: VitisNet: “Omics” Integration through Grapevine Molecular Networks
Source: PLoS One. 2009 Dec 21;4(12):e8365. doi: 10.1371/journal.pone.0008365 (PMC2791446; doi:10.1371/journal.pone.0008365)
Supplement: Text S1 — References for supporting material. (0.06 MB DOC) [file pone.0008365.s003.doc]

**Supporting Text 1: References for supporting material**

Adams-Phillips L, Barry C, Giovannoni J (2004) Signal transduction systems regulating fruit ripening. *Trends Plant Science* **9**:331–338

Aeschbacher RA, Hauser MT, Feldmann KA, Benfey PN (1995) The SABRE gene is required for normal cell expansion in *Arabidopsis*. *Genes & development* **9**:330–340.

Aubert D, Chevillard M, Dorne AM, Arlaud G, Herzog, M (1998) Expression patterns of GASA genes in *Arabidopsis thaliana*: the GASA4 gene is up-regulated by gibberellins in meristematic regions. *Plant molecular biology* **36**:871–883.

Apweiler R, Bairoch A, Wu CH, Barker WC, Boeckmann B, Ferro S, Gasteiger E, Huang H, Lopez R, Magrane M, Martin MJ, Natale DA, O'Donovan C, Redaschi N, Yeh LS (2004) UniProt: the Universal Protein knowledgebase. *Nucleic Acids Res* **32**:115-119.

Azumi Y, Liu D, Zhao D, Li W, Wang G, Hu Y, Ma H (2002) Homolog interaction during meiotic prophase I in *Arabidopsis* requires the solo dancers gene encoding a novel cyclin-like protein. *The EMBO journal* **21**:3081–3095.

Baumann K, Perez-Rodriguez M, Bradley D, Venail J, Bailey P, Jin H, Koes R, Roberts K, Martin, C (2007) Control of cell and petal morphogenesis by R2R3 myb transcription factors. *Development* **134**:1691–1701.

Ben-Naim O, Eshed R, Parnis A, Teper-Bamnolker P, Shalit A, Coupland G, Samach A, Lifschitz E (2006) The CCAAT binding factor can mediate interactions between constans-like proteins and DNA. *The Plant Journal* **46**:462–476.

Bezerra IC, Michaels SD, Schomburg FM, Amasino RM (2004) Lesions in the mRNA cap-binding gene ABA hypersensitive 1 suppress FRIGIDA-mediated delayed flowering in *Arabidopsis*. *The Plant journal* **40**:112–119.

Bowman JL, Sakai H, Jack T, Weigel D, Mayer U, Meyerowitz EM (1992) Superman, a regulator of floral homeotic genes in *Arabidopsis*. *Development* **114**:599–615.

Brocard-Gifford I, Lynch TJ, Garcia ME, Malhotra B, Finkelstein RR (2004) The *Arabidopsis thaliana* abscisic acid-insensitive8 encodes a novel protein mediating abscisic acid and sugar responses essential for growth. *The Plant cell* **16**:406–421.

Brodersen P, Petersen M, Nielsen HB, Zhu S, Newman MA, Shokat KM, Rietz S, Parker J, Mundy J (2006) *Arabidopsis* map kinase 4 regulates salicylic acid- and jasmonic acid/ethylene-dependent responses via EDS1 and PAD4. *The Plant Journal* **47**:532–546.

Cheng Y, Kato N, Wang W, Li J, Chen, X (2003) Two RNA binding proteins, HEN4 and HUA1, act in the processing of AGAMOUS pre-mRNA in *Arabidopsis thaliana*. *Developmental cell* **4**:53–66.

Chinnusamy V, Schumaker K, Zhu JK (2004) Molecular genetic perspectives on cross-talk and specificity in abiotic stress signalling in plants. *Journal of experimental botany* **55**:225–236.

Choi HI, Park HJ, Park JH, Kim S, Im MY, Seo HH, Kim YW, Hwang I, Kim SY (2005) *Arabidopsis* calcium-dependent protein kinase atCPK32 interacts with ABF4, a transcriptional regulator of abscisic acid-responsive gene expression, and modulates its activity. *Plant physiology* **139**:1750–1761.

Chung HSS, Howe GAA. (2009) A critical role for the TIFY motif in repression of jasmonate signaling by a stabilized splice variant of the jasmonate zim-domain protein JAZ10 in *Arabidopsis*. *The Plant cell* **21**:135-145.

Cockram J, Jones H, Leigh FJ, O’Sullivan D, Powell W, Laurie DA, Greenland AJ (2007) Control of flowering time in temperate cereals: genes, domestication, and sustainable productivity. *Journal of experimental botany* **58**:1231–1244.

Coego A, Ramirez V, Gil MJ, Flors V, Mauch-Mani B, Vera P (2005) An *Arabidopsis* homeodomain transcription factor, overexpressor of cationic peroxidase 3, mediates resistance to infection by necrotrophic pathogens. *The Plant cell* **17**:2123–2137.

Cole C (1998) Nucleocytoplasmic transport: Driving and directing transport. *Current Biology* **8**:368–372.

Cosgrove DJ, Li LC, Cho HT, Hoffmann-Benning S, Moore RC, Blecker D (2002) The growing world of expansins. *Plant & cell physiology* **43**:1436–1444.

Costa MM, Fox S, Hanna AI, Baxter C, Coen E (2005) Evolution of regulatory interactions controlling floral asymmetry. *Development* **132**:5093–5101.

Cui X, Fan B, Scholz J, Chen Z (2007) Roles of *Arabidopsis* cyclin-dependent kinase c complexes in cauliflower mosaic virus infection, plant growth, and development. *Plant Cell* **19**:1388–1402.

Day RB, Tanabe S, Koshioka M, Mitsui T, Itoh H, Ueguchi-Tanaka M, Matsuoka M, Kaku H, Shibuya N, Minami E (2004) Two rice GRAS family genes responsive to n-acetylchitooligosaccharide elicitor are induced by phytoactive gibberellins: evidence for cross-talk between elicitor and gibberellin signaling in rice cells. *Plant molecular biology* **54**:261–272.

Di Cola A, Klostermann E, Robinson C (2005) The complexity of pathways for protein import into thylakoids: it’s not easy being green. *Biochemical Society transactions* **33**:1024–1027.

Dill A, Thomas SG, Hu J, Steber CM, Sun TP (2004) The *Arabidopsis* f-box protein sleepy1 targets gibberellin signaling repressors for gibberellin-induced degradation. *The Plant cell* **16**:1392–1405.

Dreher K, Callis J (2007) Ubiquitin, hormones and biotic stress in plants. *Annals of Botany* **99**:787–822.

Durrant WE, Wang S, Dong X (2007) *Arabidopsis* SNI1 and RAD51D regulate both gene transcription and DNA recombination during the defense response. *Proceedings of the National Academy of Sciences of the United States of America* **104**:4223–4227.

Ellis C, Karafyllidis I, Wasternack C, Turner JG (2002) The *Arabidopsis* mutant cev1 links cell wall signaling to jasmonate and ethylene responses. *The Plant cell* **14**:1557–1566.

Francis D (2007) The plant cell cycle 15 years on. *New Phytologist* **174**:261–278.

Ge YX, Angenent GC, Wittich PE, Peters J, Franken J, Busscher M, Zhang LM, Dahlhaus E, Kater MM, Wullems GJ, Creemers-Molenaar T (2000) NEC1, a novel gene, highly expressed in nectary tissue of petunia hybrida. *The Plant journal*, **24**:725–734.

Gruenberg J, Stenmark H (2004) The biogenesis of multivesicular endosomes. *Nature reviews. Molecular cell biology* **5**:317–323.

Guo D, Gao X, Li H, Zhang T, Chen G, Huang P, An L, Li N (2008) EGY1 plays a role in regulation of endodermal plastid size and number that are involved in ethylene-dependent gravitropism of light-grown *Arabidopsis* hypocotyls. *Plant molecular biology* **66**:345–360.

Guo H, Ecker JR (2004) The ethylene signaling pathway: new insights. *Current Opinion in Plant Biology* **7**:40–49.

Guranowski A, Miersch O, Staswick PE, Suza W, Wasternack C (2007) Substrate specificity and products of side-reactions catalyzed by jasmonate:amino acid synthetase (JAR1). *FEBS letters* **581**:815–820.

Haga N, Kato K, Murase M, Araki S, Kubo M, Demura T, Suzuki K, Müller I, Voß U, Jürgens G, Ito M (2007) R1R2R3-MYB proteins positively regulate cytokinesis through activation of knolle transcription in *Arabidopsis* thaliana. *Development* **134**:1101–1110.

Hála M, Cole R, Synek L, Drdová E, Pecenková T, Nordheim A, Lamkemeyer T, Madlung J, Hochholdinger F, Fowler JE, Zárský V (2008) An exocyst complex functions in plant cell growth in *Arabidopsis* and tobacco. *The Plant cell* **20**:1330–1345.

He Y, Doyle MR, Amasino RM (2004) PAF1-complex-mediated histone methylation of flowering locus c chromatin is required for the vernalization-responsive, winter-annual habit in *Arabidopsis*. *Genes & development* **18**:2774–2784.

He Y, Gan S (2004) A novel zinc-finger protein with a proline-rich domain mediates ABA-regulated seed dormancy in *Arabidopsis*. *Plant Molecular Biology* **54**:1–9.

Hileman LC, Kramer EM, Baum DA (2003) Differential regulation of symmetry genes and the evolution of floral morphologies. *Proceedings of the National Academy of Sciences of the United States of America* **100**:12814–12819.

Himmelbach A, Hoffmann T, Leube M, Höhener B, Grill E (2002) Homeodomain protein atHB6 is a target of the protein phosphatase ABI1 and regulates hormone responses in *Arabidopsis*. *The EMBO journal* **21**:3029–3038.

Hord CL, Chen C, Deyoung BJ, Clark SE, Ma H (2006). The BAM1/BAM2 receptor-like kinases are important regulators of *Arabidopsis* early anther development. *Plant Cell* **18**:1667–1680.

Huang Z, Yeakley JM, Garcia EW, Holdridge JD, Fan JB, Whitham SA (2005) Salicylic acid-dependent expression of host genes in compatible *Arabidopsis*-virus interactions. *Plant physiology* **137**:1147–1159.

Hughes H, Stephens D (2008) Assembly, organization, and function of the COPII coat. *Histochemistry and Cell Biology* **129**:129–151.

Hugouvieux V, Murata Y, Young JJ, Kwak JM, Mackesy DZ, Schroeder JI (2002) Localization, ion channel regulation, and genetic interactions during abscisic acid signaling of the nuclear mRNA cap-binding protein, ABH1. *Plant physiology* **130**:1276–1287.

Hurley JH, Emr SD (2006) The ESCRT complexes: Structure and mechanism of a membrane-trafficking network. *Annual Review of Biophysics and Biomolecular Structure* **35**:277–298.

Hussey PJ, Ketelaar T, Deeks MJ (2006) Control of the actin cytoskeleton in plant cell growth. *Annual Review of Plant Biology* **57**:109–125.

Ishida T, Hattori S, Sano R, Inoue K, Shirano Y, Hayashi H, Shibata D, Sato S, Kato T, Tabata S, Okada K, Wada T (2007) *Arabidopsis* transparent testa GLABRA2 is directly regulated by R2R3 MYB transcription factors and is involved in regulation of GLABRA2 transcription in epidermal differentiation. *Plant Cell* **19**:2531–2543.

Jack T (2002) New members of the floral organ identity AGAMOUS pathway. *Trends in plant science* **7**:286–287.

Jaeger KE, Graf A, Wigge PA (2006) The control of flowering in time and space. *Journal of Experimental Botany* **57**:3415–3418.

Jaillon O, Aury J-M., Noel B, Policriti A, Clepet C, Casagrande A, Choisne N, Aubourg S, Vitulo N, Jubin C, Vezzi A, Legeai F, Hugueney P, Dasilva C, Horner D, Mica E, Jublot D, Poulain J, Bruyère C, Billault A, Segurens B, Gouyvenoux M, Ugarte E, Cattonaro F, Anthouard V, Vico V, Del Fabbro C, Alaux M, Di Gaspero G, Dumas V, Felice N, Paillard S, Juman I, Moroldo M, Scalabrin S, Canaguier A, Le Clainche I, Malacrida G, Durand E, Pesole G, Laucou V, Chatelet P, Merdinoglu D, Delledonne M, Pezzotti M, Lecharny A, Scarpelli C, Artiguenave F, Pè EM, Valle G, Morgante M, Caboche M, Adam-Blondon A-F., Weissenbach J, Quétier F, Wincker P (2007) The grapevine genome sequence suggests ancestral hexaploidization in major angiosperm phyla. *Nature* **449:** 463–467.

Jiang L, Xia M, Strittmatter LI, Makaroff CA (2007) The *Arabidopsis* cohesin protein SYN3 localizes to the nucleolus and is essential for gametogenesis. *The Plant Journal* **50**:1020–1034.

Jin JB, Jin YH, Lee J, Miura K, Yoo CY, Kim WY, Van Oosten M, Hyun Y, Somers DE, Lee I, Yun DJ, Bressan RA, Hasegawa PM (2008) The sumo E3 ligase, atSIZ1, regulates flowering by controlling a salicylic acid-mediated floral promotion pathway and through affects on flc chromatin structure. *The Plant Journal* **53**:530–540.

Jung JH, Seo YH, Seo PJ, Reyes JL, Yun J, Chua NH, Park CM (2007) The GIGANTEA-regulated microrna172 mediates photoperiodic flowering independent of CONSTANS in *Arabidopsis*. *Plant Cell* **19**:2736–2748.

Kakimoto T (2003) Perception and signal transduction of cytokinins. *Annual review of plant biology* **54**:605–627.

Kandasamy MK Deal RB, McKinney EC, Meagher RB (2004) Plant actin-related proteins. *Trends in plant science* **9**:196–202.

Kang HG, Foley RC, Oñate Sánchez L, Lin C, Singh KB (2003) Target genes for OBP3, a DOF transcription factor, include novel basic helix-loop-helix domain proteins inducible by salicylic acid. *The Plant journal* **35**:362–372.

Kania T, Russenberger D, Peng S, Apel K, Melzer S (1997) FPF1 promotes flowering in *Arabidopsis*. *The Plant cell* **9**:1327–1338.

Kariola T, Brader G, Helenius E, Li J, Heino P, Palva ET (2006). Early response to dehydration 15. a negative regulator of ABA-responses in *Arabidopsis*. *Plant Physiol* **142**:1559–1573.

Katsir L, Schilmiller AL, Staswick PE, He SY, Howe GA (2008) COI1 is a critical component of a receptor for jasmonate and the bacterial virulence factor coronatine. *Proceedings of the National Academy of Sciences*, **105**:7100–7105.

Kim S, Choi K, Park C, Hwang HJ, Lee I (2006) Suppressor of FRIGIDA4, encoding a C2h2-type zinc finger protein, represses flowering by transcriptional activation of *Arabidopsis* flowering locus C. *Plant Cell* **18**:2985–2998.

Kim SY, Michaels SD (2006) Suppressor of FRI 4 encodes a nuclear-localized protein that is required for delayed flowering in winter-annual *Arabidopsis*. *Development* **133**:4699–4707.

Kimura T, Nakano T, Taki N, Ishikawa M, Asami T, Yoshida S (2001) Cytokinin-induced gene expression in cultured green cells of *Nicotiana tabacum* identified by fluorescent differential display. *Bioscience, biotechnology, and biochemistry* **65**:1275–1283.

Laskowski MJ, Dreher KA, Gehring MA, Abel S, Gensler AL, Sussex IM (2002) FQR1, a novel primary auxin-response gene, encodes a flavin mononucleotide-binding quinone reductase. *Plant physiology* **128**:578–590.

Lee BH, Kapoor A, Zhu J, Zhu JK (2006) STABILIZED1, a stress-upregulated nuclear protein, is required for pre-mRNA splicing, mRNA turnover, and stress tolerance in *Arabidopsis*. *The Plant cell* **18**:1736–1749.

Lee JH, Yoo SJ, Park SH, Hwang I, Lee JS, Ahn JH (2007) Role of SVP in the control of flowering time by ambient temperature in *Arabidopsis*. *Genes & development* 21:397–402.

Li S, Assmann SM, Albert R (2006) Predicting essential components of signal transduction networks: A dynamic model of guard cell abscisic acid signaling. *PLoS Biology* 4:312.

Lim MH, Kim J, Kim YS, Chung KS, Seo YH, Lee I, Kim J, Hong CB, Kim HJ, Park CM (2004) A new *Arabidopsis* gene, FLK, encodes an RNA binding protein with k homology motifs and regulates flowering time via flowering locus C. *Plant Cell* **16**:731–740.

Liu Z, Meyerowitz EM (1995) LEUNIG regulates AGAMOUS expression in *Arabidopsis* flowers. *Development* **121**:975–991.

March-Díaz R, García-Domínguez M, Florencio FJ, Reyes JC (2007) SEF, a new protein required for flowering repression in *Arabidopsis*, interacts with PIE1 and ARP6. *Plant physiology* 143:893–901.

Mayama T, Ohtsubo E, Tsuchimoto S (2003) Isolation and expression analysis of petunia CURLY LEAF-like genes. *Plant & cell physiology* **44**:811–819.

Michaels SD, Ditta G, Gustafson-Brown C, Pelaz S, Yanofsky M, Amasino RM (2003) AGL24 acts as a promoter of flowering in *Arabidopsis* and is positively regulated by vernalization. *The Plant journal* **33**:867–874.

Miyagishima SY, Froehlich JE, Osteryoung KW (2006) PDV1 and PDV2 mediate recruitment of the dynamin-related protein ARC5 to the plastid division site. *The Plant cell* **18**:2517–2530.

Murtas G, Reeves PH, Fu YF, Bancroft I, Dean C, Coupland G (2003) A nuclear protease required for flowering-time regulation in *Arabidopsis* reduces the abundance of small ubiquitin-related modifier conjugates. *The Plant cell* **15**:2308–2319.

Naito T, Kiba T, Koizumi N, Yamashino T, Mizuno T (2007) Characterization of a unique GATA family gene that responds to both light and cytokinin in *Arabidopsis* *thaliana*. *Bioscience, biotechnology, and biochemistry* **71**:1557–1560.

Nickel W, Brugger B, Wieland FT (2002) Vesicular transport: the core machinery of COPI recruitment and budding. *Journal of Cell Science* **115**:3235–3240.

Nishimura N, Kitahata N, Seki M, Narusaka Y, Narusaka M, Kuromori T, Asami T, Shinozaki K, Hirayama T (2005) Analysis of ABA hypersensitive germination2 revealed the pivotal functions of PARN in stress response in *Arabidopsis*. *The Plant journal* **44**:972–984.

Noh B, Lee SH, Kim HJ, Yi G, Shin EA, Lee M, Jung KJ, Doyle MR, Amasino RM, Noh YS (2004) Divergent roles of a pair of homologous jumonji/zinc-finger-class transcription factor proteins in the regulation of *Arabidopsis* flowering time. *Plant Cell* **16**:2601–2613.

Paciorek T, Friml J (2006) Auxin signaling. *Journal of Cell Science* **119**:1199–1202.

Parcy F, Bomblies K, Weigel D (2002) Interaction of LEAFY, AGAMOUS and TERMINAL FLOWER1 in maintaining floral meristem identity in *Arabidopsis*. *Development* **129**:2519–2527.

Peng M, Cui Y, Bi YM, Rothstein SJ (2006) AtMBD9: a protein with a methyl-CPG-binding domain regulates flowering time and shoot branching in *Arabidopsis*. *The Plant Journal* **46**:282–296.

Pilpel Y, Segal M (2005) Rapid wave dynamics in dendritic spines of cultured hippocampal neurons is mediated by actin polymerization. *Journal of Neurochemistry* **95**:1401–1410.

Quint M, Gray W (2006) Auxin signaling. *Current Opinion in Plant Biology* **9**:448–453.

Reyes JL, Chua NH (2007) ABA induction of MIR159 controls transcript levels of two MYB factors during *Arabidopsis* seed germination. *The Plant Journal* **49**:592–606.

Riera M, Redko Y, Leung J (2006) Arabidopsis RNA-binding protein UBA2A relocalizes into nuclear speckles in response to abscisic acid. *FEBS letters* **580**:4160–4165.

Robles P, Pelaz S (2005) Flower and fruit development in *Arabidopsis thaliana*. *The International journal of developmental biology* **49**:633–643.

Rossi V, Varotto S (2002) Insights into the G1/s transition in plants. *Planta* **215**:345–356.

Saez A, Robert N, Maktabi MH, Schroeder JI, Serrano R, Rodriguez PL (2006) Enhancement of abscisic acid sensitivity and reduction of water consumption in *Arabidopsis* by combined inactivation of the protein phosphatases type 2C ABI1 and HAB1. *Plant physiology* **141**:1389–1399.

Sato T (2000) Class C VPS protein complex regulates vacuolar SNARE pairing and is required for vesicle docking/fusion. *Molecular Cell* **6**:661–671.

Schenck A, Qurashi A, Carrera P, Bardoni B, Diebold C, Schejter E, Mandel JL, Giangrande A (2004) WAVE/SCAR, a multifunctional complex coordinating different aspects of neuronal connectivity. *Developmental biology* **274**:260–270.

Schenk PM, Kazan K, Rusu AG, Manners JM, Maclean DJ (2005) The SEN1 gene of *Arabidopsis* is regulated by signals that link plant defence responses and senescence. *Plant Physiology and Biochemistry* **43**:997–1005.

Schmitz RJ, Hong L, Michaels S, Amasino RM (2005) FRIGIDA-essential 1 interacts genetically with FRIGIDA and FRIGIDA-like 1 to promote the winter-annual habit of *Arabidopsis thaliana*. *Development* **132**:5471–5478.

Segura A, Moreno M, Madueño F, Molina A, García-Olmedo F (1999) SNAKIN-1, a peptide from potato that is active against plant pathogens. *Molecular plant-microbe interactions* **12**:16–23.

Sieberer T, Hauser MT, Seifert GJ, Luschnig C (2003) PROPORZ1, a putative *Arabidopsis* transcriptional adaptor protein, mediates auxin and cytokinin signals in the control of cell proliferation. *Current biology* **13**:837–842.

Silverstone AL, Tseng TS, Swain SM, Dill A, Jeong SY, Olszewski NE, Sun TP (2007) Functional analysis of spindly in gibberellin signaling in *Arabidopsis*. *Plant physiology* **143**:987–1000.

Spoel SH, Koornneef A, Claessens SM, Korzelius JP, Van Pelt JA, Mueller MJ, Buchala, AJ, Métraux JP, Brown R, Kazan K, Van Loon LC, Dong X, Pieterse CM (2003) NPR1 modulates cross-talk between salicylate- and jasmonate-dependent defense pathways through a novel function in the cytosol. *The Plant cell* **15**:760–770.

Sridhar VV, Surendrarao A, Liu Z (2006) APETALA1 and SEPALLATA3 interact with SEUSS to mediate transcription repression during flower development. *Development* **133**:3159–3166.

Stafstrom JP, Ripley BD, Devitt ML, Drake B (1998) Dormancy-associated gene expression in pea axillary buds. cloning and expression of PSDRM1 and PSDRM2. *Planta* 205:547–552.

Stone SL, Williams LA, Farmer LM, Vierstra RD, Callis J (2006) KEEP ON GOING, a ring E3 ligase essential for *Arabidopsis* growth and development, is involved in abscisic acid signaling. *Plant Cell* **18**:3415–3428.

Swennen D, Beckerich JM (2007) *Yarrowia lipolytica* vesicle-mediated protein transport pathways. *BMC Evolutionary Biology* **7**:219

Tanaka MU, Nakajima M, Motoyuki A, Matsuoka M (2007) Gibberellin receptor and its role in gibberellin signaling in plants. *Annual Review of Plant Biology* **58**:183–198.

Telfer A, Poethig RS (1998) HASTY: a gene that regulates the timing of shoot maturation in *Arabidopsis thaliana*. *Development* **125**:1889–1898.

Teramoto H, Momotani E, Takeba G, Tsuji H (1994) Isolation of a cDNA clone for a cytokinin-repressed gene in excised cucumber cotyledons. *Planta* **193**:573–579.

The Gene Ontology Consortium (2000) Gene Ontology: tool for the unification of biology. *Nature Genet* **25***:*25-29.

Velasco R, Zharkikh A, Troggio M, Cartwright DA, Cestaro A, Pruss D, Pindo M, Fitzgerald LM, Vezzulli S, Reid J, Malacarne G, Iliev D, Coppola G, Wardell B, Micheletti D, Macalma T, Facci M, Mitchell JT, Perazzolli M, Eldredge G, Gatto P, Oyzerski R, Moretto M, Gutin N, Stefanini M, Chen Y, Segala C, Davenport C, Demattè L, Mraz A, Battilana J, Stormo K, Costa F, Tao Q, Si-Ammour A, Harkins T, Lackey A, Perbost C, Taillon B, Stella A, Solovyev V, Fawcett J A., Sterck L, Vandepoele K, Grando S M., Toppo S, Moser C, Lanchbury J, Bogden R, Skolnick M, Sgaramella V, Bhatnagar SK, Fontana P, Gutin A, Van de Peer Y, Salamini F, Viola R (2007) A high quality draft consensus sequence of the genome of a heterozygous grapevine variety. *PLoS ONE* **2:** 1326.

Wagner D, Meyerowitz EM (2002) SPLAYED, a novel SWI/SNF ATPase homolog, controls reproductive development in *Arabidopsis*. *Current biology* **12**:85–94.

Wang W, Chen X (2004) HUA ENHANCER3 reveals a role for a cyclin-dependent protein kinase in the specification of floral organ identity in *Arabidopsis*. *Development* **131**:3147–3156.

Wang ZY, Wang Q, Chong K, Wang F, Wang L, Bai M, Jia, C (2006) The brassinosteroid signal transduction pathway. *Cell Research* **16**:427–434.

Wasteneys GO, Yang Z (2004) New views on the plant cytoskeleton. *Plant physiology* **136**:3884–3891.

Woodward AW, Bartel B (2005) Auxin: Regulation, action, and interaction. *Annals of Botany* **95**:707–735.

Woodward C, Bemis SM, Hill EJ, Sawa S, Koshiba T, Torii KU (2005) Interaction of auxin and erecta in elaborating *Arabidopsis* inflorescence architecture revealed by the activation tagging of a new member of the YUCCA family putative flavin monooxygenases. *Plant physiology* **139**:192–203.

Xiong L, Lee H, Ishitani M, Tanaka Y, Stevenson B, Koiwa H, Bressan RA, Hasegawa PM, Zhu JK (2002) Repression of stress-responsive genes by FIERY2, a novel transcriptional regulator in *Arabidopsis*. *Proceedings of the National Academy of Sciences of the United States of America* **99**:10899–10904.

Yang CH, Chen LJ, Sung ZR (1995) Genetic regulation of shoot development in arabidopsis: role of the EMF genes. *Developmental biology* 169:421–435.

Yoshida N, Yanai Y, Chen L, Kato Y, Hiratsuka J, Miwa T, Sung ZR, Takahashi S (2001) Embryonic flower2, a novel polycomb group protein homolog, mediates shoot development and flowering in *Arabidopsis*. *The Plant cell* **13**:2471–2481.

Yoshida R, Umezawa T, Mizoguchi T, Takahashi S, Takahashi F, Shinozaki K (2006) The regulatory domain of SRK2E/OST1/SNRK2.6 interacts with ABI1 and integrates abscisic acid (ABA) and osmotic stress signals controlling stomatal closure in *Arabidopsis*. *The Journal of biological chemistry* **281**:5310–5318.

Zhang X, Garreton V, Chua NH (2005) The AIP2 E3 ligase acts as a novel negative regulator of ABA signaling by promoting ABI3 degradation. *Genes & development* **19**:1532–1543.

Zhao D, Yang M, Solava J, Ma H (1999) The ASK1 gene regulates development and interacts with the UFO gene to control floral organ identity in *Arabidopsis*. *Developmental genetics* **25**:209–223.
