# Supplementary material for: VitisNet: “Omics” Integration through Grapevine Molecular Networks
Source: PLoS One. 2009 Dec 21;4(12):e8365. doi: 10.1371/journal.pone.0008365 (PMC2791446; doi:10.1371/journal.pone.0008365)
Supplement: Text S2 — Tutorial for Using VitisNet, a database for the grapevine molecular networks. (3.51 MB DOC) [file pone.0008365.s004.doc]

**Supporting Text 2**

**Tutorial for Using VitisNet, a database for the Grapevine Molecular Networks**

**This tutorial is also available at:** [**http://vitis-dormancy.sdstate.org**](http://vitis-dormancy.sdstate.org/) **in the VitisNet tab.**

**Introduction:**

VitisNet ([vitis-dormancy.sdstate.org](http://vitis-dormancy.sdstate.org/)) has been built with a program called CellDesigner ([celldesigner.org](../../../../G:/celldesigner.org)) and can be visualized using Cytoscape ([cytoscape.org](../../../../G:/cytoscape.org)). A user first identifies a set of genes, transcripts, proteins or metabolites of interest and then can overlay their expression values on the molecular networks (VitisNet) to determine biochemical events of interest. Additional plug-ins available in Cytoscape can be used to analyze the data further. The following tutorial will instruct users on what software is needed, how to obtain that software and how to upload data and visualize them on the molecular networks of [VitisNet](http://vitis-dormancy.sdstate.org/pathways.cfm). A set of data has been provided in the right hand panel of the [VitisNet](http://vitis-dormancy.sdstate.org/pathways.cfm) page that can be used with this tutorial.

Please Note: The networks are works in progress: in cooperation with the scientific community’s curation of gene annotations, we are planning to update existing and release new networks at MetNet (<http://metnet3.vrac.iastate.edu/>) and VitisNet ([http://vitis-dormancy.sdstate.org](http://vitis-dormancy.sdstate.org/)).  Individuals may submit edits in the form of cell designer SBML files to [METNET](http://metnet3.vrac.iastate.edu/), [Jérôme Grimplet](mailto:jerome.grimplet@icvv.es) ,or [Anne Fennell](mailto:anne.fennell@sdstate.edu). Edits in the form of as description may be submitted to [Jérôme Grimplet](mailto:jerome.grimplet@icvv.es) or [Anne Fennell](mailto:anne.fennell@sdstate.edu) for implementation.

**Description of VitisNet network files:**

The networks are available in several different formats at VitisNet (<http://vitis-dormancy.sdstate.org/pathways.cfm>). The user does not need to work with all of these file types. The different file types provide flexibility of access and some work better with different operating systems.

1. Preview: for browsers that just want to look at the molecular netWorks. Simply click on preview to view any one of the 220 molecular networks. A static pdf image of the individual network in CellDesigner ([celldesigner.org](../../../../G:/celldesigner.org)) format will be displayed.
2. SBML: Network files in SBML format. Individual files can be opened with CellDesigner or imported into Cytoscape with the BiNoM Plugin (see section 2.1 “Uploading CellDesigner files” and section 8). The tutorial example outlined in sections 2-6 uses SBML file type. The networks were created in CellDesigner and users wishing to edit networks will find it easier to use these files.
3. XGMML: Network files in XGMML format. Individual files can be imported directly into Cytoscape without a plugin (See section 2.2). McIntosh users may find these files easier to use.
4. Cytoscape Session - Raw Networks: Network files preprocessed with the BiNoM plugin. All files have been assembled into six functional groups of molecular networks (*.cys files) that can be downloaded and used in Cytoscape or a webstart version of Cytoscape (section 7.2). The Raw Network files allow user to load expression data onto several pathways at once. It does have some limitations in processing networks for clarifying the network view, as indicated in section 4.1 (converting nodes to edges). However, users can view their own data on several molecular networks at once without processing the individual networks, thus providing a rapid survey of biochemical events (section 7.1). After surveying their data users can always make their own functional groupings as indicated in Section 4.1.
5. Cytoscape Session - Networks with molecular abundance in berry tissues: Network files preprocessed with the BiNoM plugin. All files have been assembled into six functional groups of molecular networks (*.cys files) with berry expression data inserted. These can be opened using Cytoscape software on user computer or using a webstart version of Cytoscape (section 7.2 ) to view the data from [Grimplet et al. (2007)](http://www.biomedcentral.com/1471-2164/8/187) and [Grimplet et al. (2009)](http://dx.doi.org/10.1002/pmic.200800158).

The molecular networks are also available for browsing at MetNet (<http://metnet3.vrac.iastate.edu/>).

**Using VitisNet with Cytoscape**

1. The user should first download the Cytoscape software and the BiNoM Plugin into a folder on their computer (Section 1).
2. Download and install Cytoscape (<http://cytoscape.org/>). Download BiNoM (<http://bioinfo-out.curie.fr/projects/bionom/>) and place the file (BiNoM_all.jar) in the plugins folder located in the Cytoscape program folders.
3. To use tutorial download four files from the right hand panel of the VitisNet tab ([GS_to_probeset.txt](http://vitis-dormancy.sdstate.org/images/vitis_img/GS_to_probeset.txt), [expression_berry.txt](http://vitis-dormancy.sdstate.org/images/vitis_img/expression_berry.txt), [berrystyle.props](http://vitis-dormancy.sdstate.org/images/vitis_img/berrystyle.props), Citrate cycle (TCA Cycle), (SBML file: vv10020CitrateCycle.xml) to a local folder. Right click on the file and save as target to your local folder. Macintosh users need to “ctrl right click” and "Download linked file as" to save it to a specific local folder.

Please note that file downloads work best using Mozilla Firefox. If you use Internet Explorer and any file that you download does not have the file type ending that is designated in this tutorial, the browser may have changed the file type. You may try changing these erroneous file types back to the file type indicated in this tutorial (for example if a file downloads as a *.zip change it to the correct file type of *.jar or *.cys). Alternatively use the Firefox browser to access and download files and Cytoscape software. Laptop users will want to use a mouse with two buttons and a scroll wheel (for ease of zooming in and out of networks).

**1. Installation of the Required Software**

**1.1 Cytoscape (mandatory for tutorial):**

Cytoscape (<http://www.cytoscape.org/>) is the freely available software required for visualizing VitisNet and loading expression data. Go to the download area (1) and follow the instructions. New Cytoscape users should follow the Cytoscape tutorials available on the Cytoscape website (2) [http://cytoscape.org/cgi-bin/moin.cgi/Presentations/Basic.](http://cytoscape.org/cgi-bin/moin.cgi/Presentations/Basic) It is highly recommended to complete tutorials 01 and 04 to understand the flexibility of Cytoscape and to facilitate using VitisNet networks and loading and using expression data.


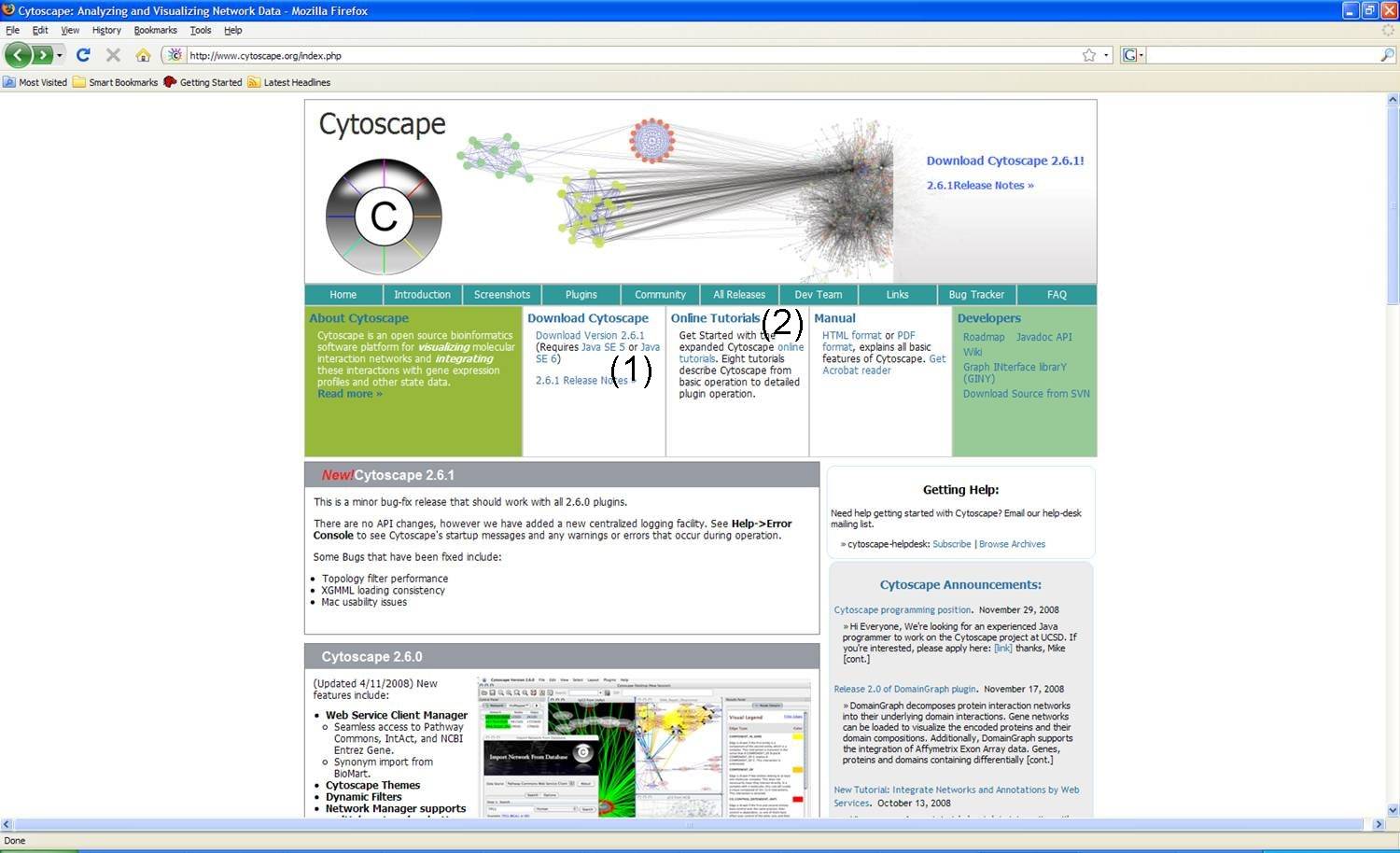


**1.2 BiNoM plugin (mandatory for tutorial):**

BiNoM is a Cytoscape plugin that allows the importation of SBML files (including CellDesigner files) into Cytoscape. The BiNoM plugin is freely available at <http://bioinfo-out.curie.fr/projects/binom/>. Go to the download area (1), click on Download, 22M and save. Be sure that the file name is BiNoM_all.jar. If it is BiNoM_all.zip change it to .jar and change the drop down “save as type” to All Files and then save to a local folder. Once downloaded, this plugin needs to be copied into the plugins folder in your local Cytoscape directory.

Note: Macintosh users have had problems with the BiNoM plugin, they can skip this and use XGMML files in the tutorial. The XGMML files do not need the BiNoM plugin.

**
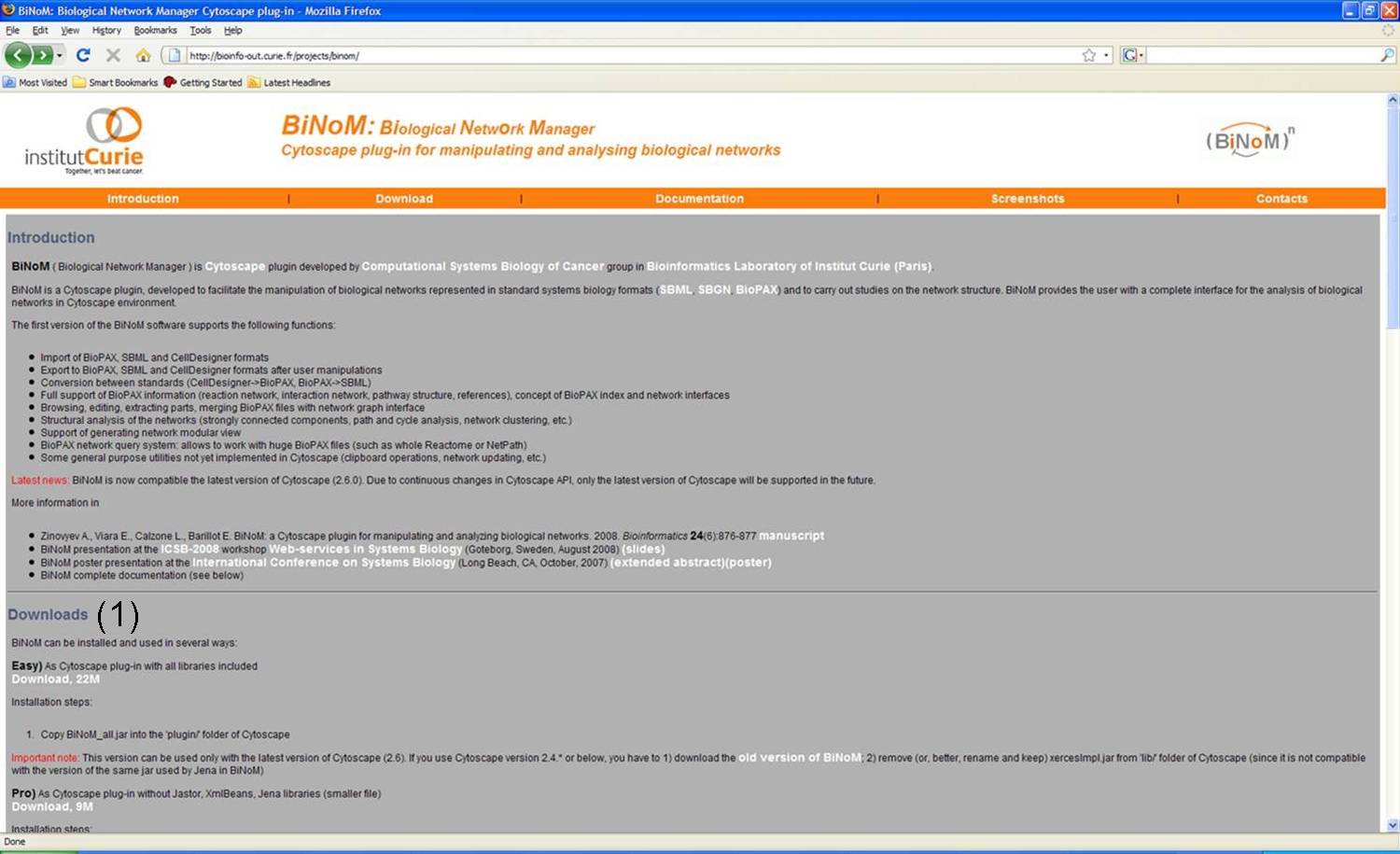
**

**2. Loading a Network into Cytoscape**

Once Cytoscape and BiNoM have been installed and the tutorial files have been downloaded to a local folder, open Cytoscape. This tutorial uses berry sample data and one of the CellDesigner SBML files (Citrate Cycle (TCA Cycle) from the main VitisNet tab to illustrate the steps for uploading and working with VitisNet files in Cytoscape. Only one file format needs to be used for this tutorial or any other work. Uploading different file types found on VitisNet site are also described, however the user does not need to download all types. Macintosh users may want to start with XGMML files (Section 2.2) instead of SBML Networks as problems have been found with the BiNoM plugin on Macintosh computers.

**2.1** **Uploading SBML Networks:**

To upload SBML files created with CellDesigner, go to the Plugins menu of Cytoscape and choose "BiNoM I/O > Import CellDesigner Document from file". Go to the local folder that contains the downloaded network sbmL file(s) and select vv10020CitrateCycle.xml, which should appear in an XML format. Select "Open". A status window will appear. Once the network is open, the status window can be closed.


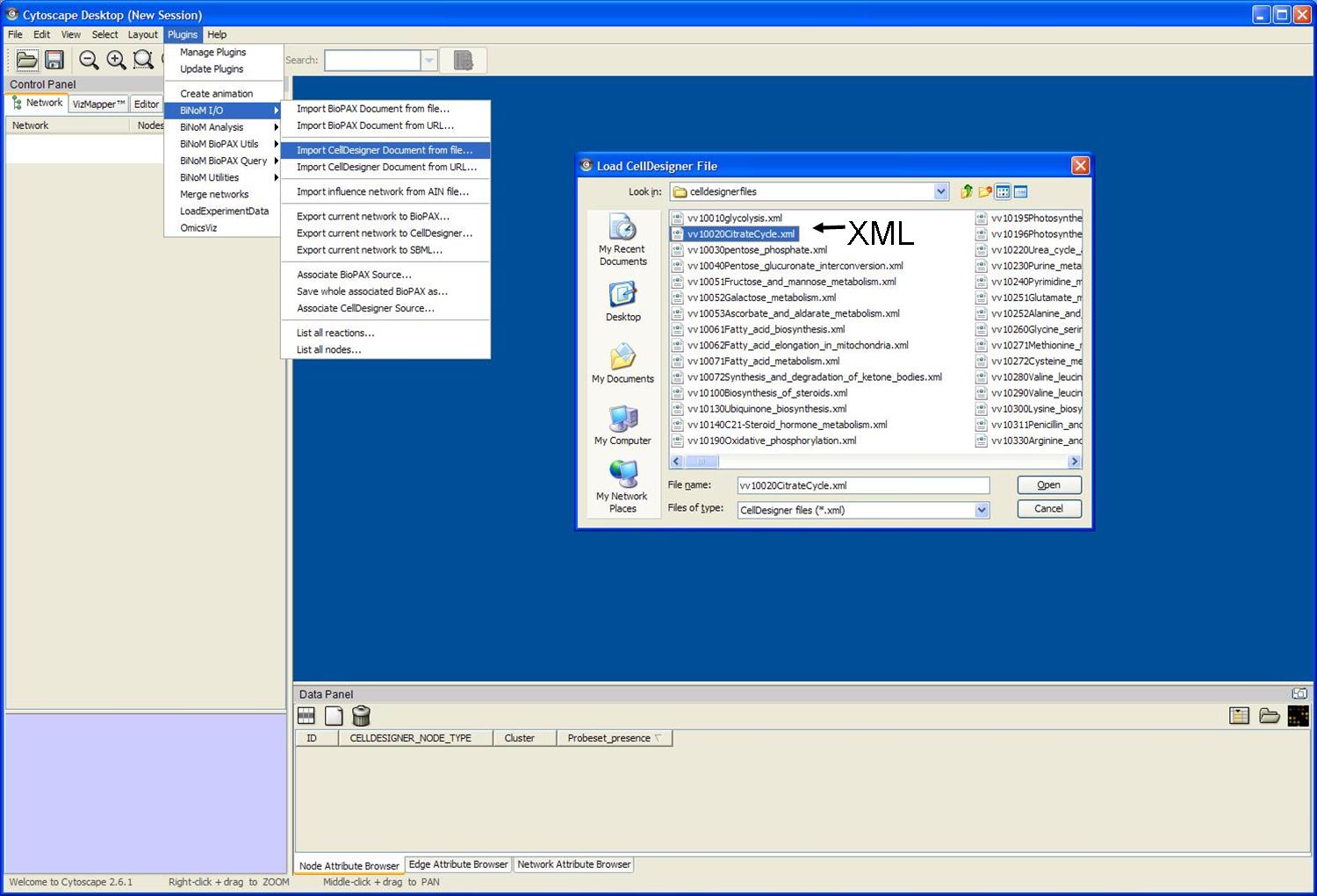


- 1. **Uploading XGMML:**

Macintosh users should use XGMML files which do not require the BioNoM Plugin. To upload the XGMML files (third column in the networks directory on VitisNet webpage), go to the File menu and choose "Import > Network (multiple file types)". The "Import Network" window should open. Click on “Select” and pick a file that is in the XGMML format. Click “Import” back in the “Import Network” window. A status window should open confirming that the network is loaded. This status window can be closed.

Note: Macintosh users need to “ctrl right click” and "Download linked file as" to save it to a specific local folder.


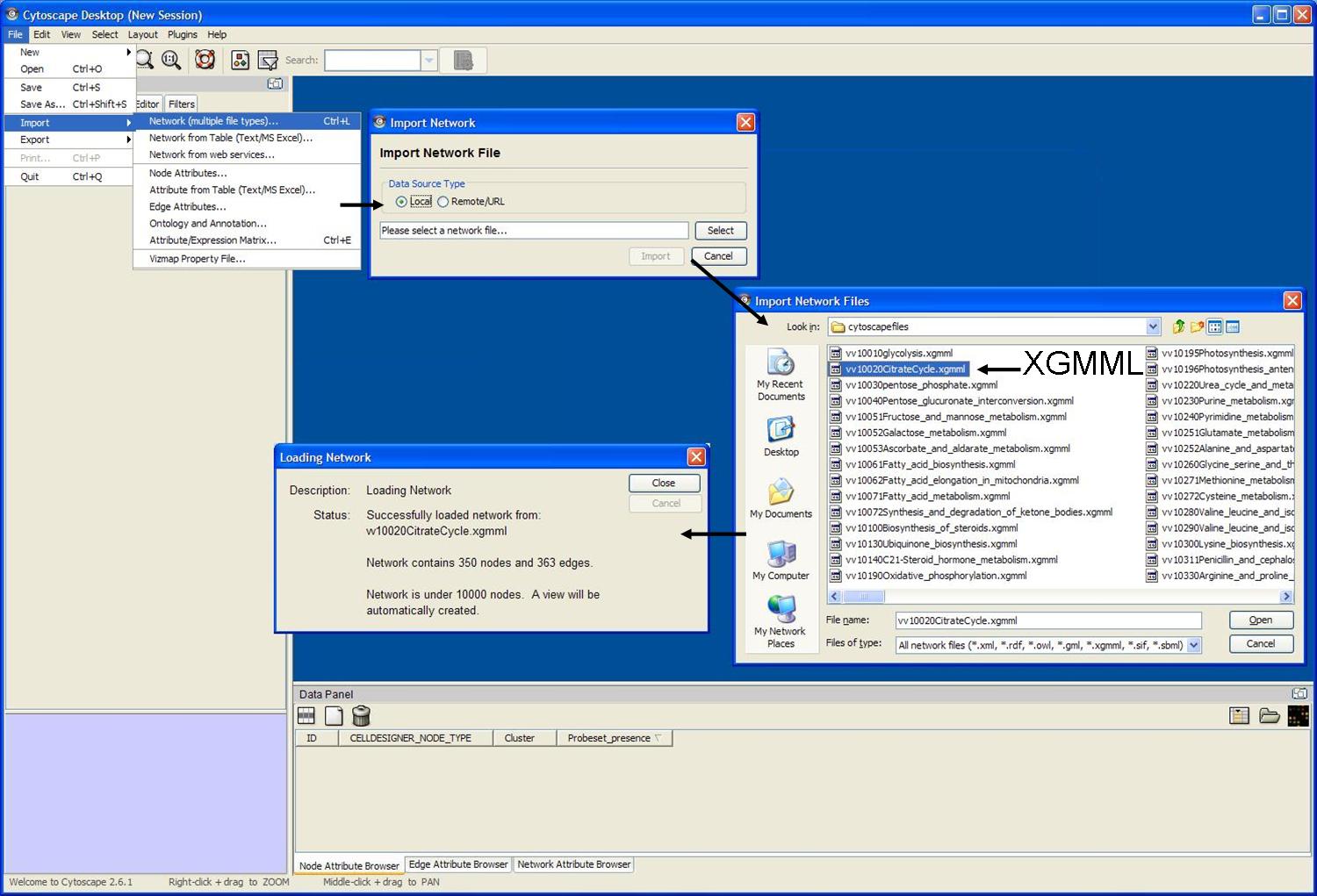


# 3. Visual Style and Viewing Networks

When the network file(s) are loaded (section 2.1 or 2.2), the networks appear under the BiNoM CellDesigner visual style or a style with the name of the network (XGMML file). The properties of the styles are accessible under the Vizmapper tab in the Control Panel on the left side of Cytoscape. However these visual styles do not always provide the best layout to view the VitisNet. It is important to understand how to load and modify visual style. The visual style allows users to customize how the networks are displayed. It is also used for visualizing expression data by modifying the color of the nodes according to the expression of the molecule that they represent.

**3.1 Uploading visual style file:**

To upload the visual properties files into Cytoscape, go into the File menu and choose "Import > Vizmap Property File". The "Import Visual Property File" window will open. Go to the local folder where you have downloaded the [berrystyle.props](http://vitis-dormancy.sdstate.org/images/vitis_img/berrystyle.props) file, and click to open it in Cytoscape. A status window will appear, which can be closed.


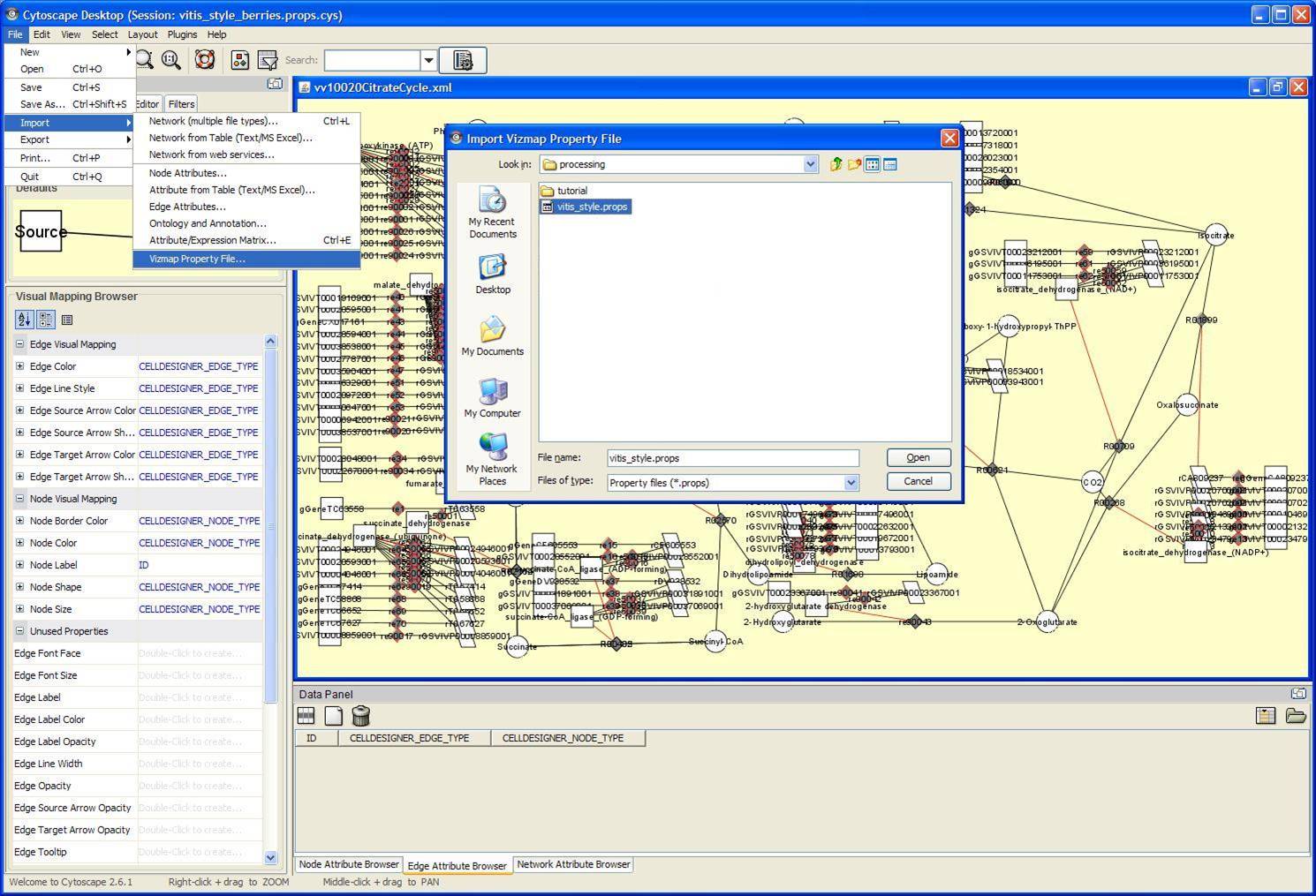


**Note:** If you do not see text associated with nodes as seen in the above diagram you will need to edit “preferences”. In the edit drop down, go to preferences and click on properties. A “Cytoscape Preference Editor” box will appear. In the property box, scroll down to “render.nodeLabelThreshold” click modify and a modify threshold box will appear. In this box, set the value to 600 or higher and click OK. If you cannot see text associated with the nodes, you will need to experiment (My PC is set at 20,000, Macintosh is set at 600). Keep increasing this value to find one that works well for your screen.

**3.2 Changing the visual style:**

Once a visual style has been uploaded, it is available through the VizMapper tab (1). Select it from the "Current Visual Style" drop down menu (2). You can choose from a variety of different styles that are built into Cytoscape, but select Vitis_styleberry.


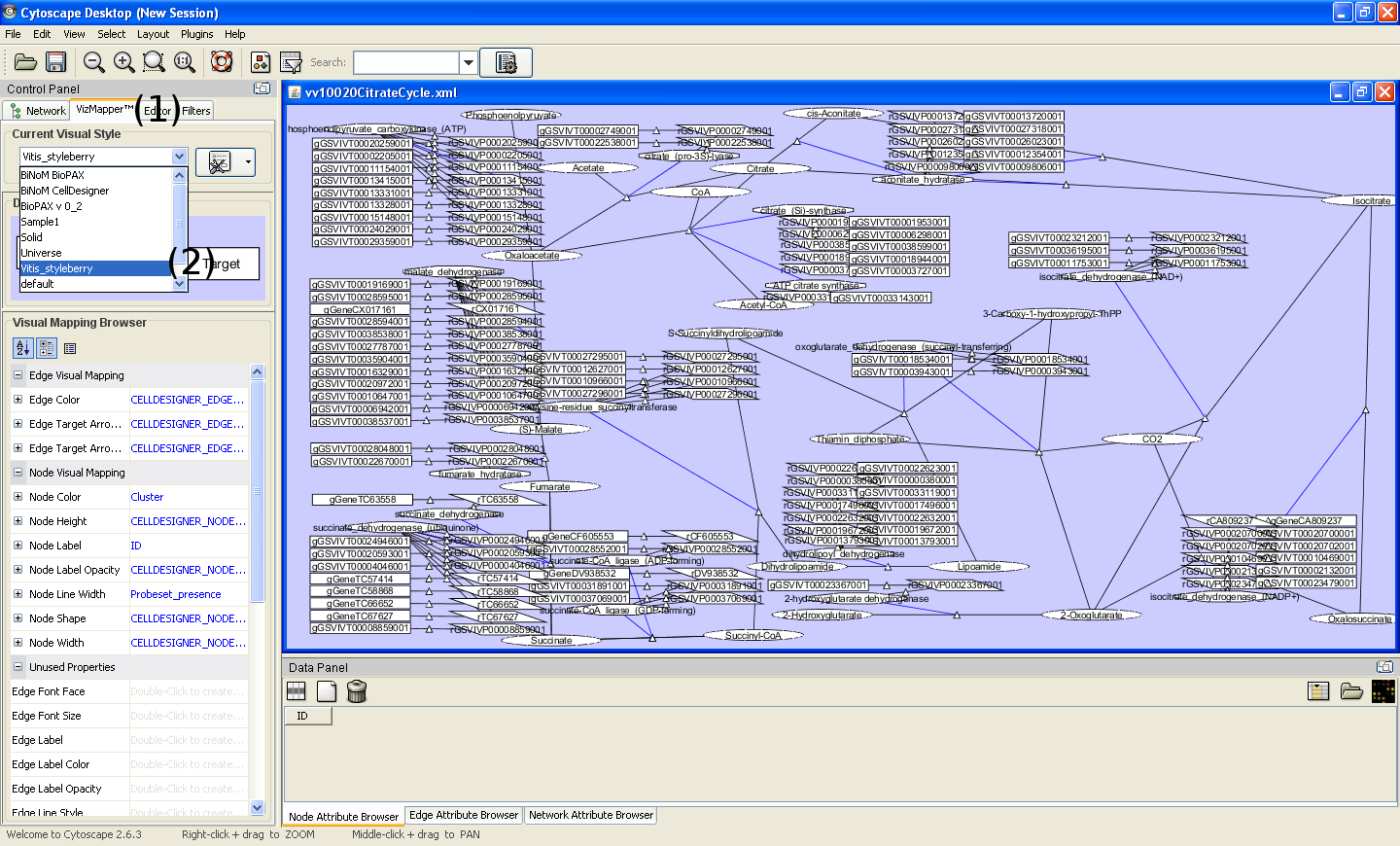


**3.3 Visual style legend:**

This picture lists the descriptions of the nodes and edges styles from the Vitis_styleberry that are used in the example, but users can experiment and adjust style to their liking as they become familiar with Cytoscape. The edge and node styles for Vitis-Stleberry are accessible in the “Visual Mapping Browser” section of the Control Panel on the left side of the screen. The descriptions of these styles are accessed by expanding the menu (+) of the elements. Alternatively, users can click on the “Defaults” picture above the “Visual Mapping Browser” selecting node, edge or global tab.


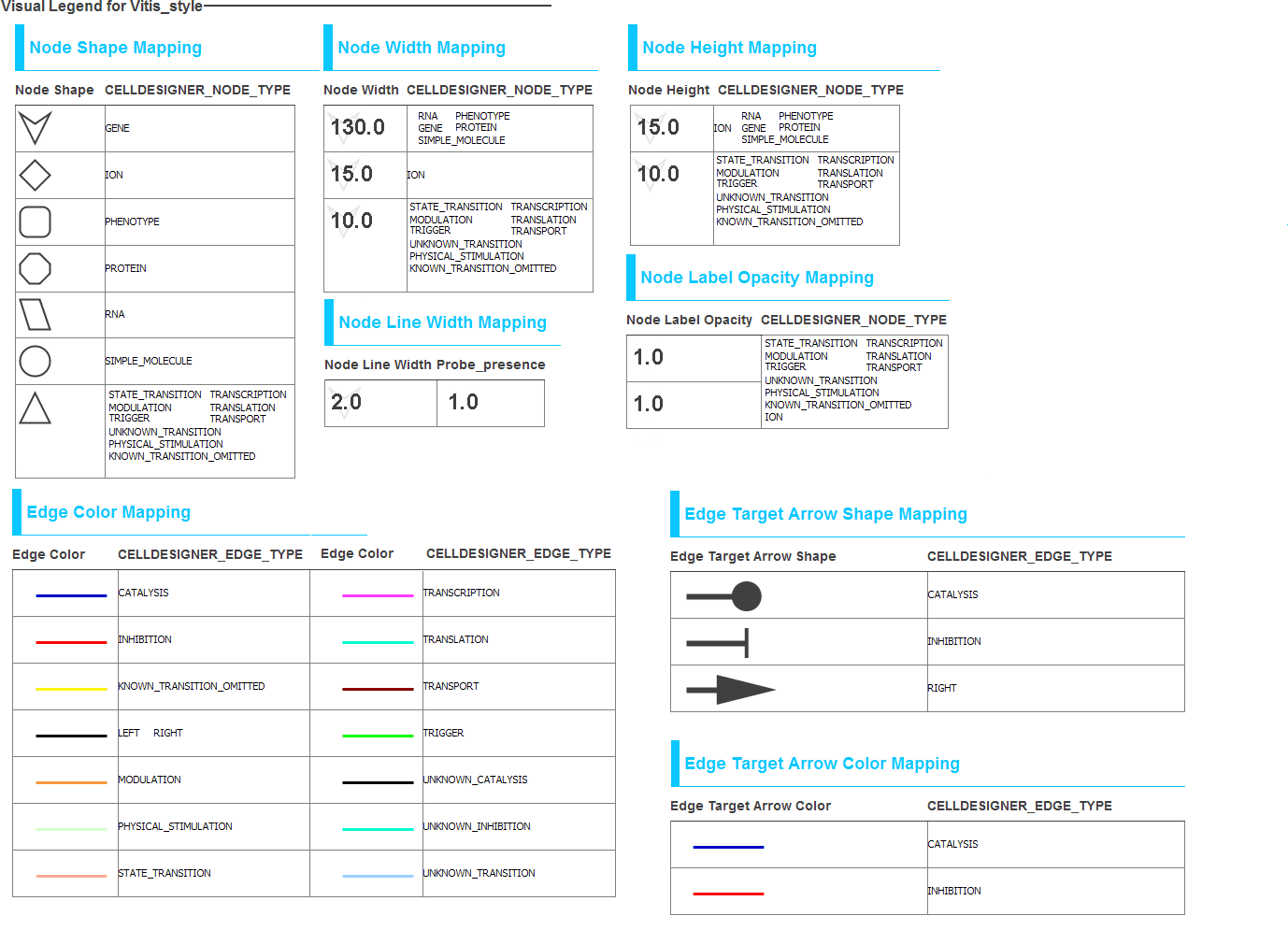


For example, background color can be changed by clicking on the “Defaults” image, going to the global tab, clicking on “background color” and picking white (or any other color in the global tab). Font attributes can be changed by clicking on the “source” or “target” box in “Defaults” and making adjustments. Consult the Cytoscape tutorial at Cytoscape.org for more customization details.


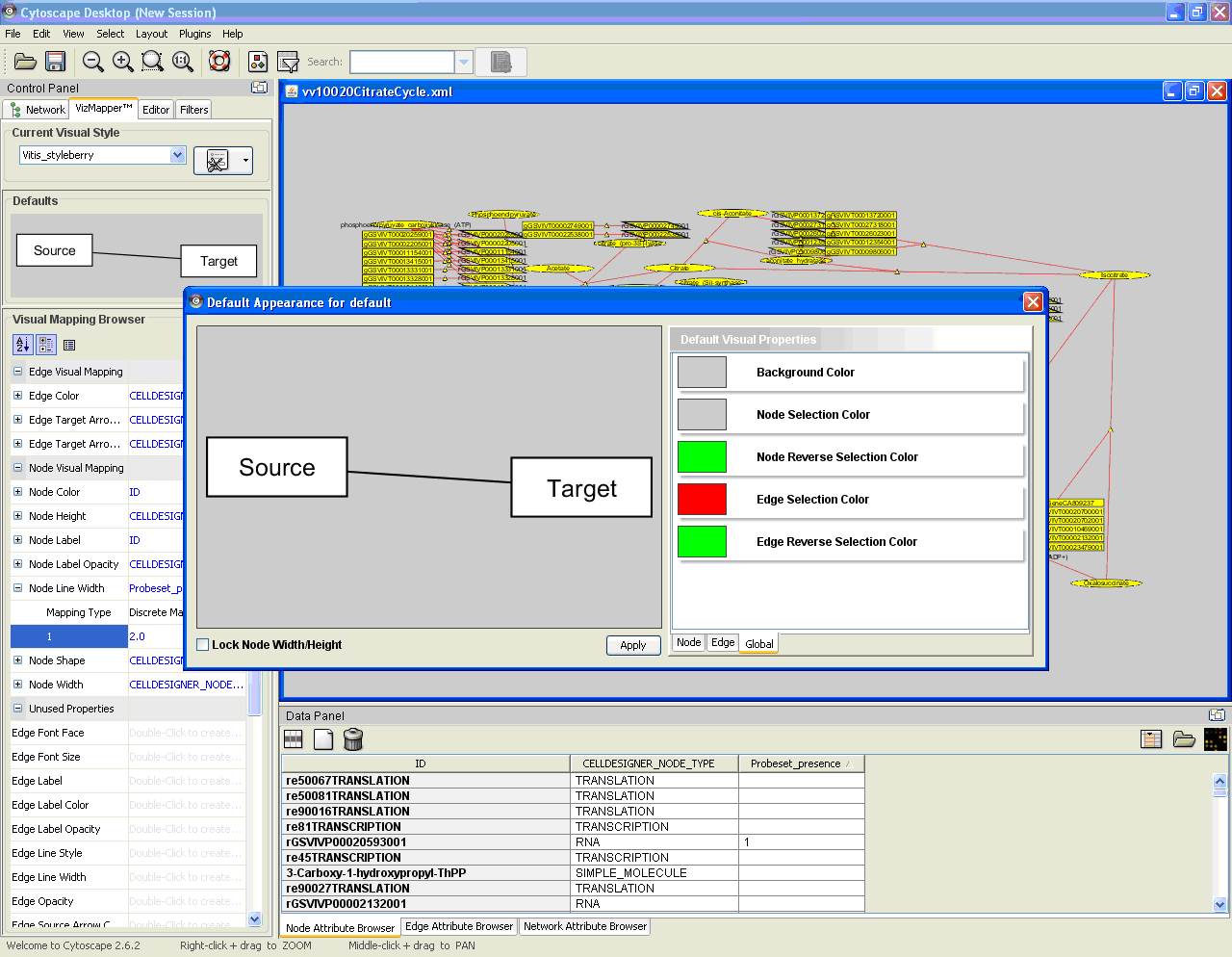


1. **Switching the Reaction from Nodes to Edges**

This section is useful for increasing the clarity of visualization of the networks, but it is not crucial. The “Vitis_styleberry" visual style allows users to view distinct edge colors for each type of reaction. However, when the BiNoM plugin converts SBML files into Cytoscape readable format, it converts all the edges into nodes. Instead of having one edge that links molecule A to molecule B, now there is one edge that links molecule A to a reaction node that is linked to molecule B with a second edge. This is necessary when a reaction involves more than 2 molecules, as is the case for enzymatic reactions (ie: at least 3 nodes, a substrate, a product and an enzyme). It does not occur when a reaction is represented that involves only 2 molecules, such as a transcription or translation event. BiNoM allows you to convert these types of reactions back from nodes to edges.

Note this does not need to be done with XGMML files.

**4.1 Converting nodes to edges:**

In the plugins menu, select "BiNoM analysis > Mono-molecular reaction to edges". A status window will appear, which can be closed.


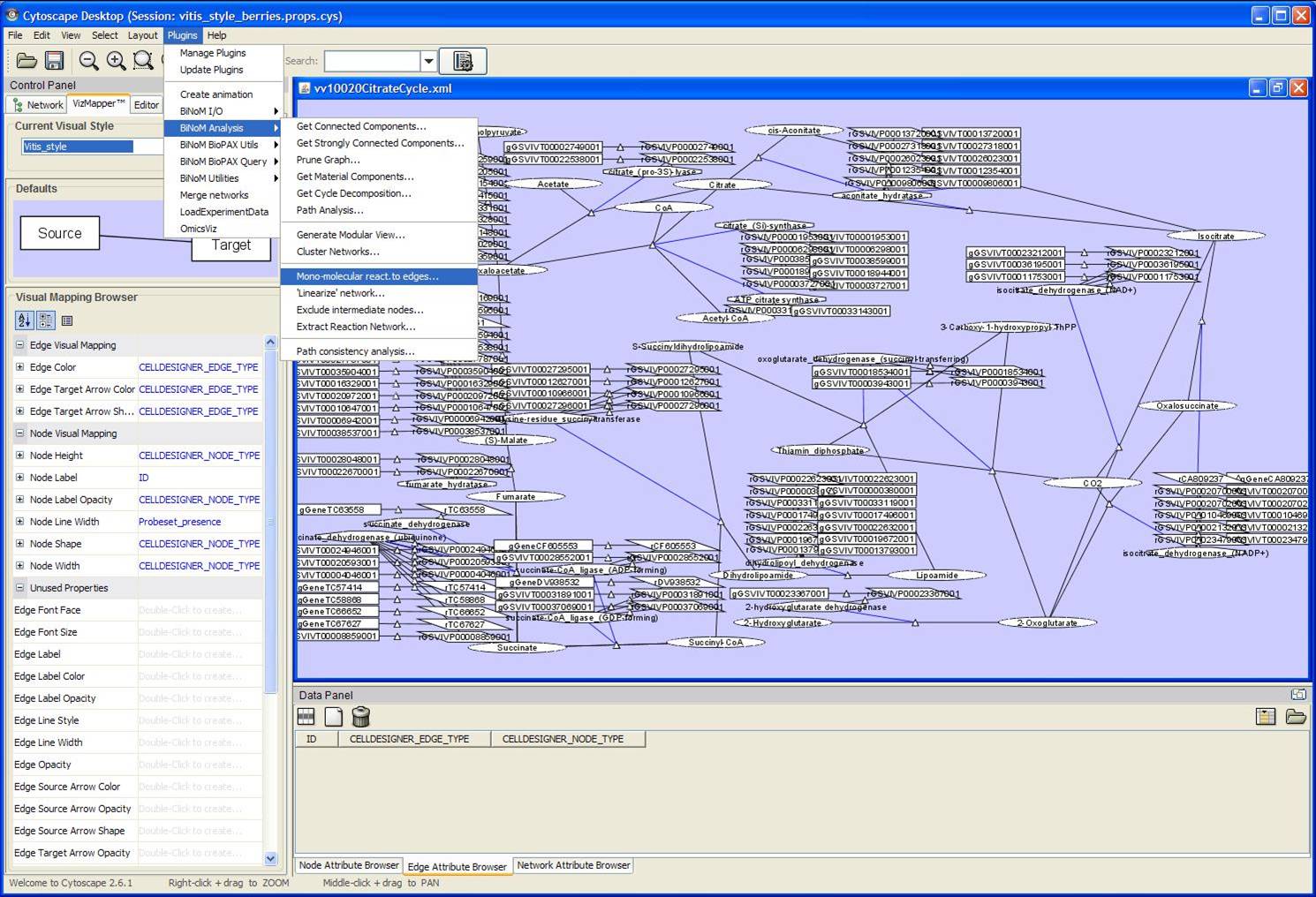


**4.2 Setting attributes:**

The mono-molecular reactions nodes have been converted into edges but their « type » is not in the same attribute class as the edge types that did not require modification. All the types need to be regrouped under the same class, the CELLDESIGNER_EDGE_TYPE, for viewing reaction-specifics colors. Go to the "Edge Attribute Browser" tab in the "Data panel" frame (1). Select the whole network by clicking and dragging on the image (all the nodes should appear in yellow and the edges in red). Click on the "Select Attributes" icon (2) and check CELLDESIGNER_EDGE_TYPE and CELLDESIGNER_NODE_TYPE. The first one is the original edge type attribute class and the second one has been created during the conversion from nodes to edges. Click outside of box to close it. The attributes from the NODE_TYPE need to be moved into the EDGE_TYPE. Click on the "Attribute Batch Editor" icon (4), and window will open. Go to the "Copy" tab (5) and select CELLDESIGNER_NODE_TYPE in the first drop down menu and CELLDESIGNER_EDGE_TYPE in the second one. Click on “Go”. Close the box once the action is done. Click anywhere on network to deslect it. Edge colors are now visible on the networks and accessible through the Vizmapper Vitis styleberry (6).


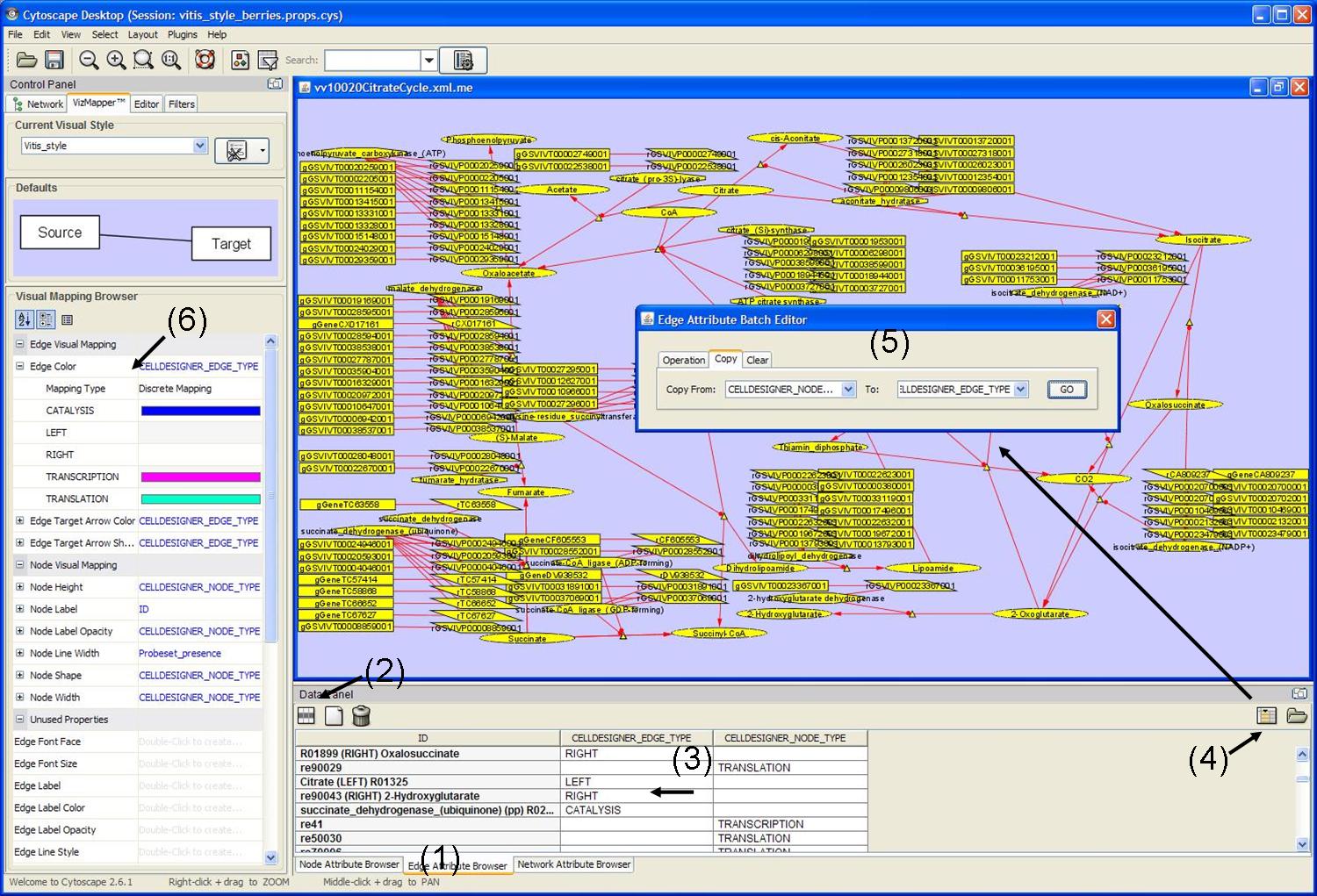


1. **Probeset Attributes**

This section describes how to link microarray features to the unique transcript sequences used in the networks. In the following example data from Affymetrix GeneChip® *Vitis vinifera* Genome Array hybridizations has been used. If your microarray directly links expression to the gene name used in the molecular networks this step can be skipped.

**5.1 Loading probesets attributes:**

Loading expression data in Cytoscape is equivalent to assigning any attribute to the items present on the networks. In this example for transcriptomics data, the steps for uploading microarray data obtained from Affymetrix GeneChips are described. A preliminary step is needed to link the probeset IDs to the transcripts. Although several Affymetrix probesets can be related to each individual transcript, there are up to twelve probesets that correspond to one transcript. The probeset with the highest expression across all grapevine microarray experiments in Plexdb has been listed as the first probeset in the first column in the GS_to_probeset.txt file. In the File menu go to "Import > Attribute from Table". The "Import Annotation File" window will open. Load the GS_to_probeset.txt file (1) in the "Data Sources" section. In the "Advanced" section check the "Show Mapping Options" (2) and the "Show Text File Import Options" (3) boxes. In the "Annotation File to Attribute Mapping" section, select CELLDESIGNER_SPECIES as "Key Attribute for Network" (4), as it is the attribute present in both data sets, i.e. the transcript name. In the "Attribute Names" section, check the "Transfer first line as attribute names" (5) box. Click “Import” (located at the very bottom of the window). Close the status window. If users want to map transcripts from another species or grapevine genes with different IDs, this procedure can be used by replacing the probeset name with the orthologous gene name or ID.


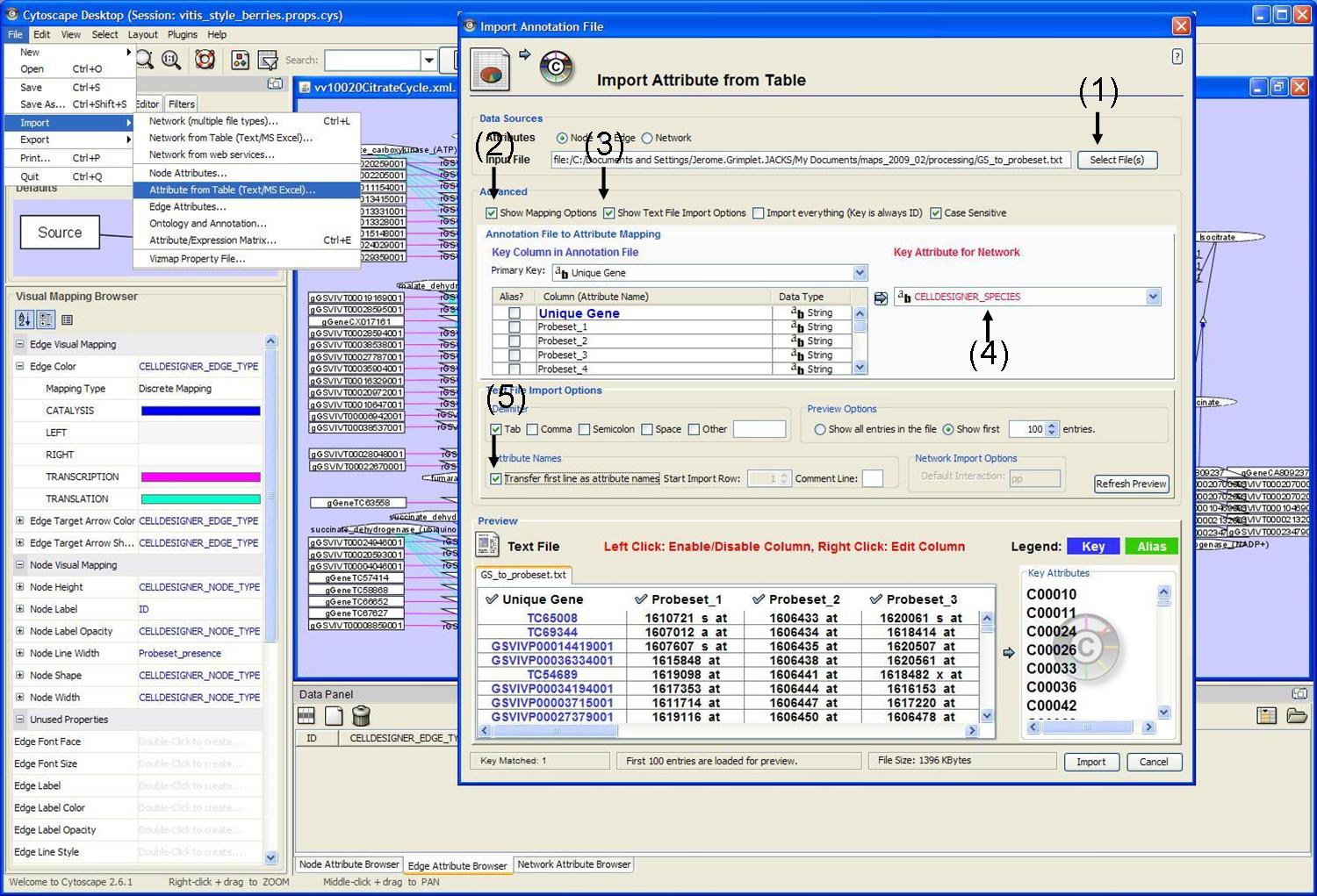


**5.2 Visualizing probesets:**

Once the data are loaded, the borders of the transcript nodes that contain a probeset appear with a bold outline (1). You may need to zoom in a little bit to see the bold clearly. However, if the transcripts are not bolded like in the picture below, click on node line width in the visual mapper browser (3). Click and select Discrete Mapper. “1” appears below “Mapping Type”. Click in the cell under “discrete Mapping” and type “2”. Click enter on keyboard. Each transcript with a probeset is flagged in the Probeset_presence node attribute column (2) in the Data Panel at the bottom of the frame. If the probeset presence column contains a 1, then the node line thickness with it is multiplied by 2 (3). For visualizing attributes in the bottom frame, click on Node Attribute browser tab; select items from network by clicking and dragging cursor over desired section; and use the “Select Attributes” icon (see section 4.2) to select desired section; and information will appear in Data Panel (i.e. CELLDESIGNER_NODE_TYPE AND probeset_presence).


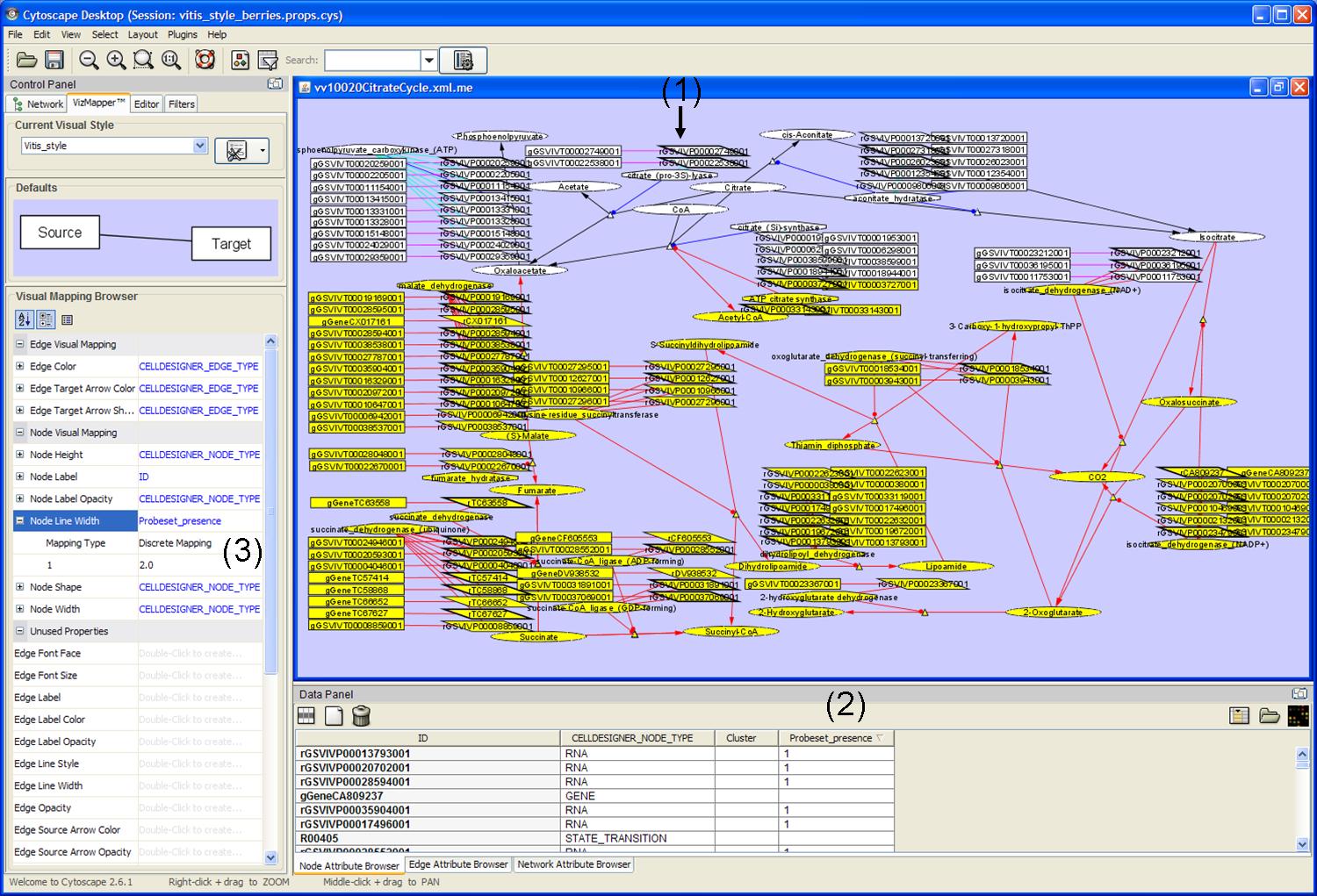


1. **Expression Data**

This section describes how to load expression data from transcriptomic, proteomic and metabolomic studies.

**6.1 Loading expression data:**

The expression data (expression_berry.txt) can now be assigned to the transcript via the probeset attribute. This section describes how to load metabolite, protein and transcript expression values. In the File menu go to "Import > Attribute from Table". The "Import Annotation File" window will open. Load the expression file expression_berry.txt (1) in the "Data Sources" section. In the "Advanced" section check the "Show Mapping Options" (2) and the "Show Text File Import Options" (3) boxes. In this experiment, the metabolites and the proteins are directly mapped to the CELLDESIGNER_SPECIES, which are KEGG metabolite numbers and EC numbers or identifiers for the proteins. In the "Annotation File to Attribute Mapping" section, select CELLDESIGNER_SPECIES as "Key Attribute for Network" (4). In the "Attribute Names" section, check the "Transfer first line as attribute names" (5) and click “Import”. The transcript expression values are mapped to the probeset attribute. Next repeat the steps above with the probeset_1 as "Key Attribute for Network" (4) to complete mapping expression description/value to probeset. If you want to map expression to the probesets 2 to 12 repeat that operation to your discretion.


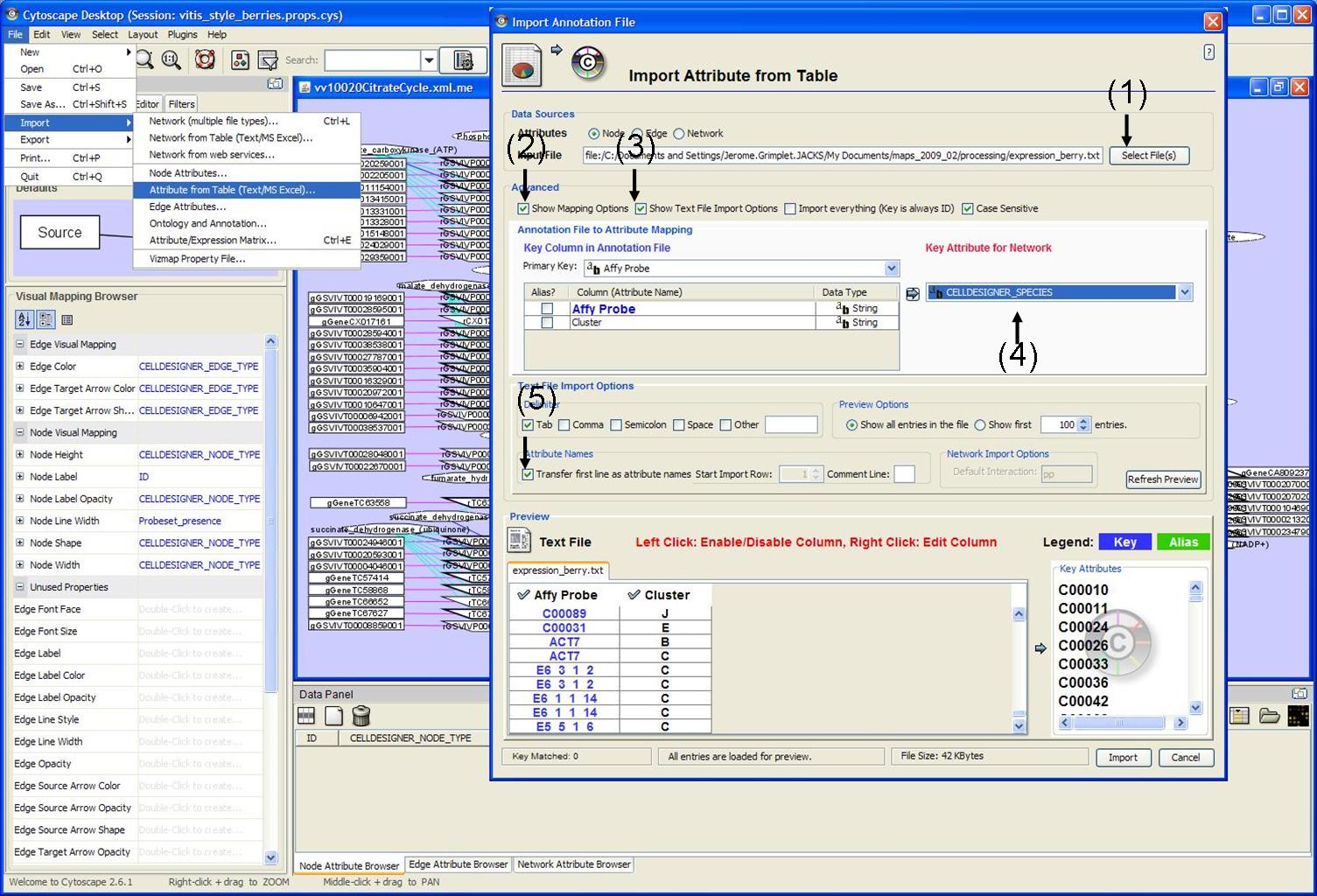


**Note:** For using your own expression data, users need to create a text file that contains in the first column an ID that can map to an attribute present on the networks. It can be a main ID, referenced here as “CELLDESIGNER_SPECIES”. It can also be an intermediate, such as the probeset ID as used in the tutorial for the transcripts. The next column(s) would contain your expression data. For this tutorial, data were clustered, and each expression pattern was represented with a letter in only one column. However any type of data can be uploaded, including expression values for each condition in one column. The Node Color attribute in Vizmapper can be modified accordingly. The visual style is highly customizable and any kind of attribute can be modified according to the expression.

**6.2 Visualizing expression data:**

When the expression data are loaded, nodes corresponding to metabolites, transcripts and proteins with differential abundance appear with colors representing their respective abundance. Sometimes the colors don’t appear when the networks are loaded. Click on Node color and Discrete mapping. The colors will need to be redefined. Only the expression clusters present on the pathway that you have opened will be visible, not necessarily the 12 possible clusters.


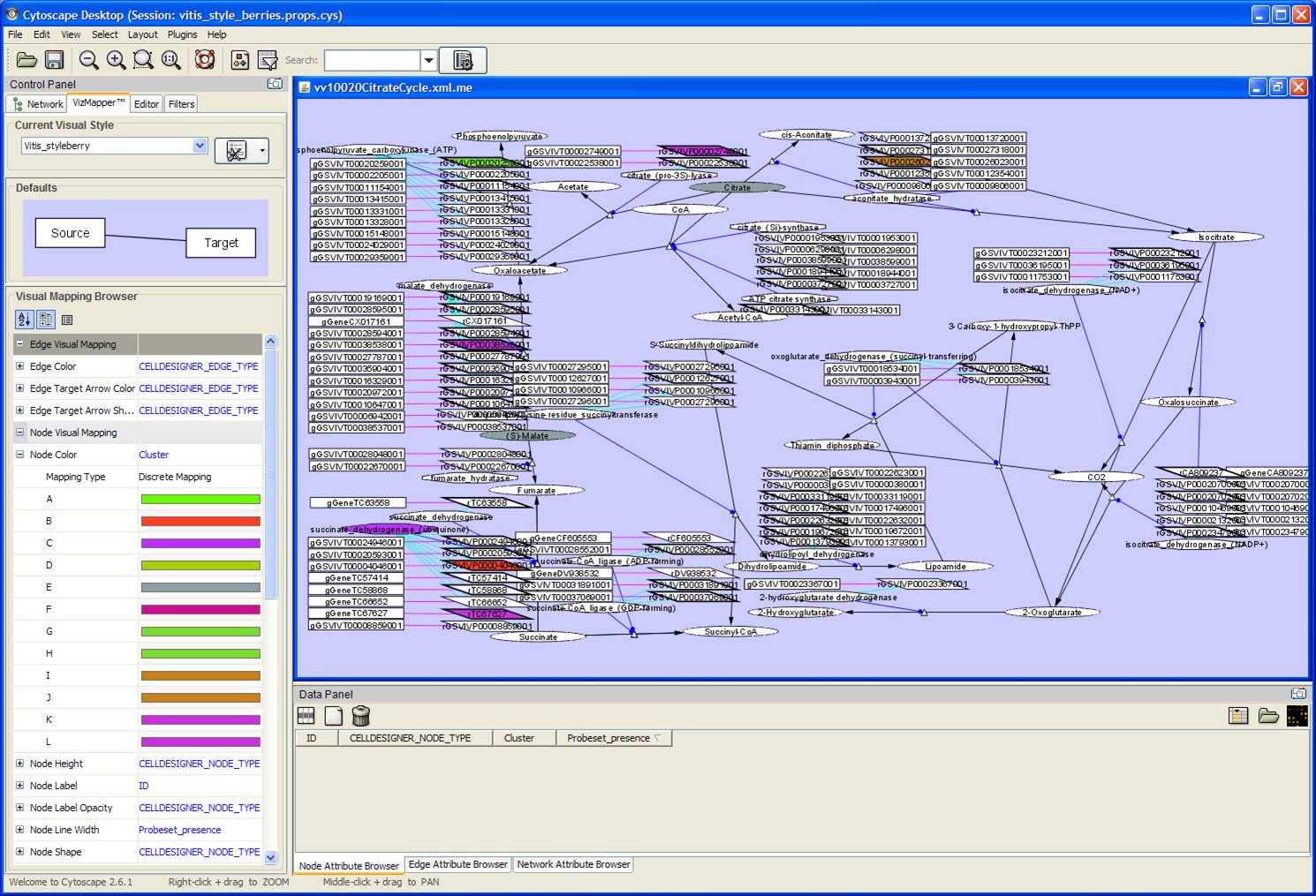


1. **Using Preprocessed Cytoscape Sessions and Cytoscape Webstart**

Networks processed with the BiNoM plugin are available for a quick start to viewing data.

1. Raw networks: can be used to quickly overlay data for the entire VitisNet (see section 6).
2. Networks with molecular abundance in berry: provides a set of preprocessed networks that already have berry expression data (Grimplet et al. (2007) and Grimplet et al. (2009) loaded into them.

Download the *.cys files from the right hand panel of the VitisNet tab (vitis-dormancy.sdstate.org) to a local folder and open in Cytoscape. Networks with molecular abundance in berry sessions do not need any processing, simply open the files. If using Raw Networks with your own data, follow instructions in Sections 2, 3, 4.2, 5 and 6. Do not try converting nodes and edges (Section 4.1) as this should only be done with a single file, save the converted file and reimport as XGMML. BiNoM doesn’t properly handle reactions with identical ID in different networks. If you wish to group networks (as has been done with the Cytoscape Sessions used here), process one network and save it, then open and process the second network. To group networks, export the networks as XGMML and re-import them together as described in section 2.2. (For exporting click on file>export>Export Network and Attributes as XGMML and save your file as .xgmml.)

If you do not wish to download Cytoscape software you may view these files by using a webstart version of Cytoscape (<http://www.cytoscape.org/tut/webstart/cyto.jnlp> or at <http://bioinformatics.psb.ugent.be/cytoscape/>). When webstart Cytoscape is open, Go to file>open, go to the local folder that the networks were downloaded to, chose one Cytoscape session and click on open.

** Please note, this will give you a quick view. If you plan to work with your own data it is better to follow the tutorial and place Cytoscape on your local computer.

1. **CellDesigner (optional)**

Installation of CellDesigner is not required, however it is helpful for visualization of the networks or if you wish to edit networks. The networks were built with CellDesigner and the positions of the items within the networks are suited for visualization with that software. In Cytoscape, the items may shift. CellDesigner is freely available at <http://www.celldesigner.org/>. Go to the download area (1) and follow the instructions.

**
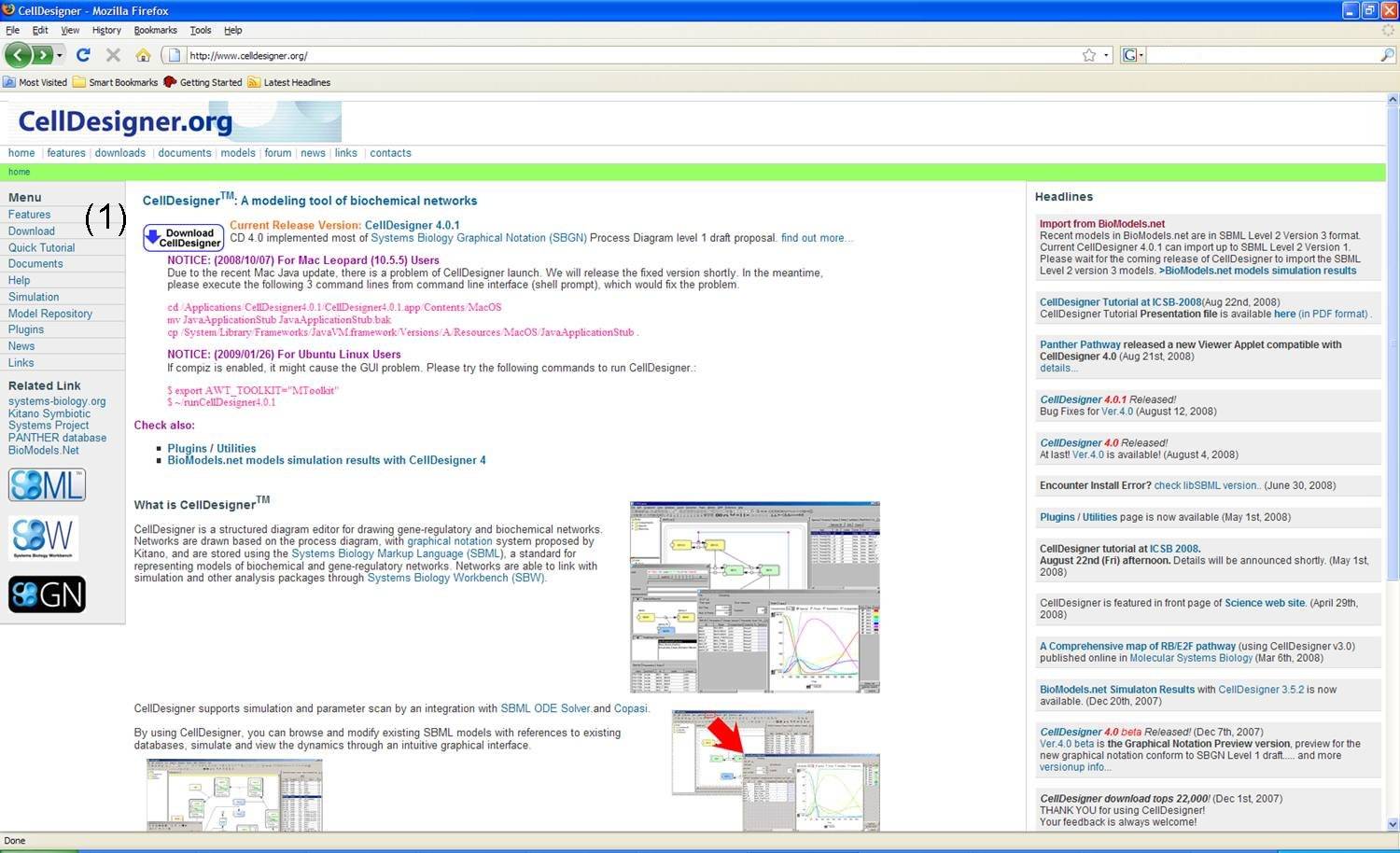
**

Literature Citations

Grimplet J, Deluc LG, Tillett RL, Wheatley MD, Schlauch KA, Cramer GR, Cushman JC (2007) Tissue-specific mRNA expression profiling in grape berry tissues. BMC Genomics 8: 187.

Grimplet J, Wheatley MD, Ben Jouira H, Deluc LG, Cramer GR, Cushman JC (2009) Proteomic and selected metabolite analysis of grape berry tissues under well watered and water-deficit stress conditions. Proteomics DOI: 10.1002/pmic.200800158.
